# Multi-omics reveals that the rumen microbiome and its metabolome together with the host metabolome contribute to individualized dairy cow performance

**DOI:** 10.1186/s40168-020-00819-8

**Published:** 2020-05-12

**Authors:** Ming-Yuan Xue, Hui-Zeng Sun, Xue-Hui Wu, Jian-Xin Liu, Le Luo Guan

**Affiliations:** 1 Institute of Dairy Science, Ministry of Education Key Laboratory of Molecular Animal Nutrition, College of Animal Sciences, Zhejiang University, Hangzhou, 310058 China; 2 Department of Agricultural, Food and Nutritional Science, University of Alberta, Edmonton, AB T6G 2P5 Canada

**Keywords:** Dairy cattle, Milk protein yield, Rumen metagenome, Rumen metabolome, Serum metabolome

## Abstract

**Background:**

Recently, we reported that some dairy cows could produce high amounts of milk with high amounts of protein (defined as milk protein yield [MPY]) when a population was raised under the same nutritional and management condition, a potential new trait that can be used to increase high-quality milk production. It is unknown to what extent the rumen microbiome and its metabolites, as well as the host metabolism, contribute to MPY. Here, analysis of rumen metagenomics and metabolomics, together with serum metabolomics was performed to identify potential regulatory mechanisms of MPY at both the rumen microbiome and host levels.

**Results:**

Metagenomics analysis revealed that several *Prevotella* species were significantly more abundant in the rumen of high-MPY cows, contributing to improved functions related to branched-chain amino acid biosynthesis. In addition, the rumen microbiome of high-MPY cows had lower relative abundances of organisms with methanogen and methanogenesis functions, suggesting that these cows may produce less methane. Metabolomics analysis revealed that the relative concentrations of rumen microbial metabolites (mainly amino acids, carboxylic acids, and fatty acids) and the absolute concentrations of volatile fatty acids were higher in the high-MPY cows. By associating the rumen microbiome with the rumen metabolome, we found that specific microbial taxa (mainly *Prevotella* species) were positively correlated with ruminal microbial metabolites, including the amino acids and carbohydrates involved in glutathione, phenylalanine, starch, sucrose, and galactose metabolism. To detect the interactions between the rumen microbiome and host metabolism, we associated the rumen microbiome with the host serum metabolome and found that *Prevotella* species may affect the host’s metabolism of amino acids (including glycine, serine, threonine, alanine, aspartate, glutamate, cysteine, and methionine). Further analysis using the linear mixed effect model estimated contributions to the variation in MPY based on different omics and revealed that the rumen microbial composition, functions, and metabolites, and the serum metabolites contributed 17.81, 21.56, 29.76, and 26.78%, respectively, to the host MPY.

**Conclusions:**

These findings provide a fundamental understanding of how the microbiome-dependent and host-dependent mechanisms contribute to varied individualized performance in the milk production quality of dairy cows under the same management condition. This fundamental information is vital for the development of potential manipulation strategies to improve milk quality and production through precision feeding.

Video Abstract

## Background

Meeting the demand for animal protein products has become a primary global food security concern as the world population continues to increase [1]. Dairy milk is an indispensable high nutritional animal protein product, and the annual global per capita dairy consumption is over 100 kg/year [2]. Many factors can affect dairy cow milk production and quality including genetics [3], management [4], and feed strategy [5]. It has been widely reported that milk yield is usually negatively correlated with milk protein content [6]. However, we have found that some dairy cows can produce both high milk yield and high milk protein content comparing to others when they were fed the same diet and under the same management [7]. We defined this as milk protein yield (MPY, high milk protein content × high milk yield or low milk protein content × low milk yield), which can be a potential new trait selected for dairy producers [7].

The rumen serves as a bioreactor that enables dairy cows to obtain nutrients from human-indigestible plant mass, and we speculated that rumen microbiome can directly and indirectly affect host MPY. Indeed, we found that several rumen bacterial taxa contributed to the milk yield and milk components [8], and different rumen bacterial richness and compositional patterns were observed between cows with high and low MPY [7]. However, recent studies have highlighted that even when the rumen microbiomes had differential taxonomic compositions, the metabolic functions were similar [9], suggesting that the difference in the microbiota at the composition and taxonomic levels may not be directly associated with its metabolic functions that affect the host. Although identifying metabolic functions of the rumen microbiome is vital, the metabolic functions of the rumen microbiome reported to date are largely based on metagenomics [10] and/or metatranscriptomics [11–13] without integrating metabolomics to investigate the metabolic-level functions of the microbiome. Therefore, we further hypothesized that the rumen microbiome in high MPY cows has different rumen microbial metabolites compared with those of low MPY cows, leading to varied MPY phenotypes.

In addition, recent studies have also reported that the rumen microbiome, together with the host, affected methane emission [14] and feed efficiency [15] in dairy cows. The biosynthesis of milk in dairy cows is a complicated biological process that involves not only the rumen but also host metabolic processes. The milk production and biosynthesis of milk protein in dairy cows is a complicated biological process that involves not only the rumen but also host metabolic processes. For example, the serum metabolome analysis revealed that 36 metabolites had different abundances between high and low MPY cows [6], indicating that host metabolism can indeed contribute to MPY biological processes. For milk protein biosynthesis, the dietary crude protein is firstly degraded and the degraded protein is then utilized to synthesize the microbial protein in the rumen, which together with undegraded dietary protein is digested into amino acids and absorbed in the small intestines. The amino acids are transported to the liver and then transported into the mammary gland through the bloodstream for the synthesis of milk protein [16]. Therefore, we further hypothesized that the rumen microbiome and its metabolites could affect the host metabolism (reflected by the serum metabolome), and subsequently affect the MPY. In this study, we performed rumen metagenomics, rumen metabolomics, and serum metabolomics on dairy cows with significantly different MPY to address the following fundamental questions: do the rumen microbiome (composition and functions), microbial metabolites, and the host metabolites contribute to MPY? If so, do they affect this trait equally? The rumen microbiome and metabolome, as well as the host metabolome, were compared between dairy cows with high and low MPY, and the contributions of the above three omics layers to MPY were calculated. The current study will provide fundamental information about the microbiome-dependent and host metabolome-dependent mechanisms that contribute to high-quality dairy milk production.

## Results

### Characterization of phenotypes

In this study, previously reported milking traits were obtained from 374 dairy cows [8], and 10 cows with the highest MPY (cows with high milk yield and milk protein content; HH) and 10 cows with the lowest MPY (cows with low milk yield and milk protein content; LL) were selected for metagenome, rumen metabolome, and serum metabolome analyses. Among the phenotypes, milk yield (*P* < 0.01), milk protein content (*P* < 0.01), and MPY (*P* < 0.01) were significantly different between the HH and LL groups (Table S1).

### Profiling of the rumen metagenome

Metagenome sequencing generated a total of 1,069,431,480 reads, with 66,839,468 ± 1,168,990 reads (mean ± standard error of the mean [SEM]) per sample (Table S2). After quality control and removing host genes, a total of 1,033,603,420 reads were retained, with 64,600,214 ± 1,165,364 per sample. After de novo assembly, a total of 12,097,293 contigs were generated (the N50 length of 795 ± 28 bp), with 756,081 ± 27,721 per sample. The rumen metagenome consisted of 94.43% bacteria (355,456,488 sequences), 3.80% eukaryotes (14,312,486 sequences), 1.41% archaea (5,292,432 sequences), and 0.16% viruses (601,612 sequences; Figure S1).

**Fig. 1:**
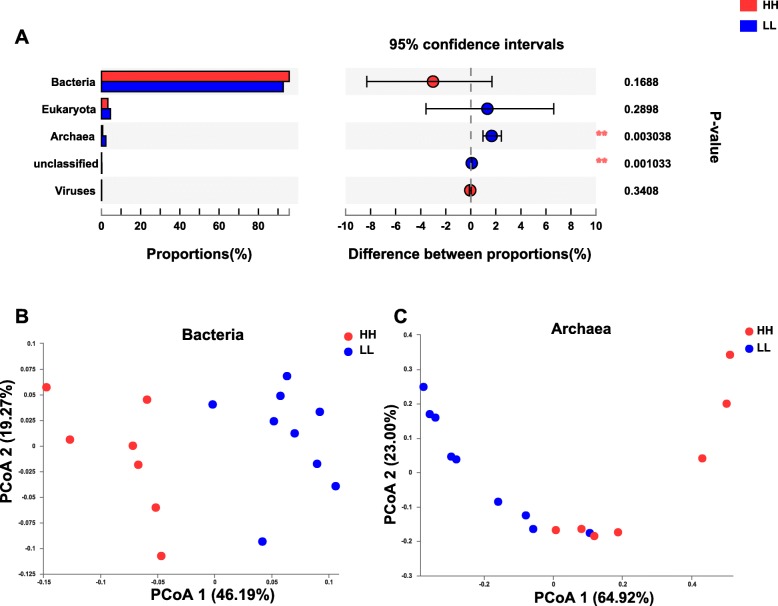
Microbial compositional profiles of HH and LL cows. **a** Comparison of microbial domains between HH and LL cows. Significantly different domains were tested by Wilcoxon rank-sum test with adjusted *P* value of < 0.05. ** *P* < 0.01. **b** Bacterial compositional profiles of HH and LL rumen samples based on species visualized using principal-coordinate analysis (PCoA). **c** Archaeal compositional profiles of HH and LL rumen samples based on species visualized using PCoA

The microbial domains were compared between the rumen microbiomes of the two MPY groups, and archaea were significantly different between the two groups (adjusted *P* < 0.01, Fig. 1a). The permutational multivariate analysis of variance (PERMANOVA) showed that both bacteria and archaea were significantly different (adjusted *P* < 0.01), while eukaryota and viruses were not different (adjusted *P* > 0.05) between the two groups (Table S3). The principal coordinate analysis (PCoA) showed separations between the two MPY groups based on bacterial (Fig. 1b) and archaeal species (Fig. 1c), while no separation was found based on eukaryotic or viral species (Figure S2). Thus, the downstream comparison of rumen microbial taxa between the two groups of animals was focused only on bacteria and archaea.

### Compositional profiles of the rumen microbiome and taxonomic differences between the HH and LL cows

**Fig. 2:**
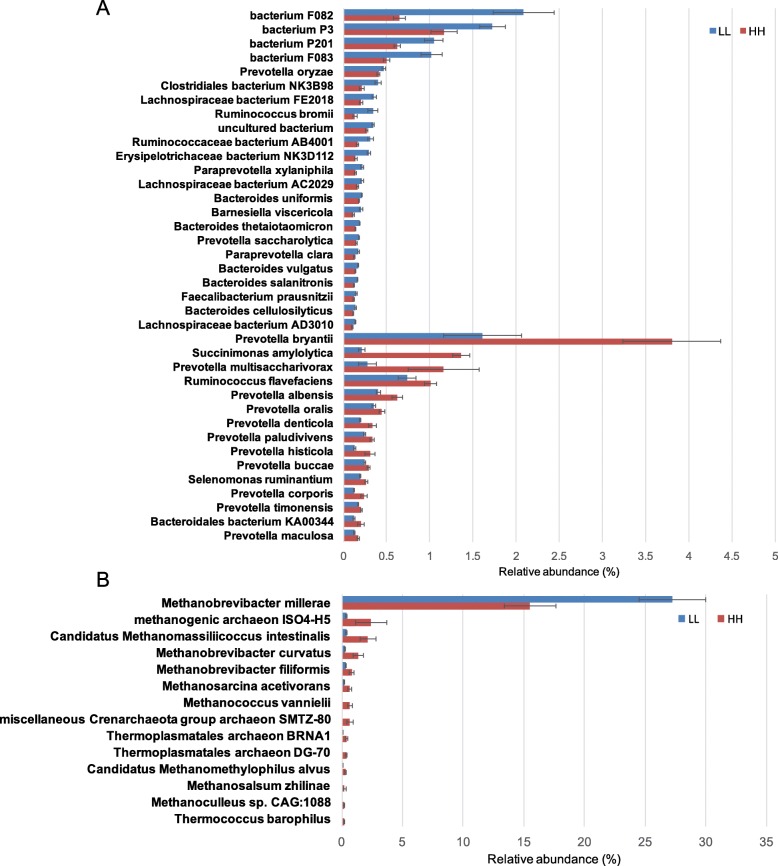
Differential rumen bacterial and archaeal species between HH and LL cows. **a** Significantly different bacterial species. **b** Significantly different archaeal species. Significant differences were tested by linear discriminant analysis effect size (LEfSe) analysis, with linear discriminant analysis (LDA) score of > 2 and *P* value of < 0.05

The dominant bacterial phyla included *Bacteroidetes* (55.98 ± 1.02%), *Firmicutes* (27.32 ± 1.14%), and *Proteobacteria* (7.32 ± 1.57%); the dominant bacterial genus was *Prevotella* (41.95 ± 0.85%), followed by *Bacteroides* (7.29 ± 0.31%), unclassified *Lachnospiraceae* (3.29 ± 0.18%), and *Clostridium* (2.99 ± 0.19%); and the dominant bacterial species included *Prevotella* sp. FD3004 (7.01 ± 0.37%), *Prevotella ruminicola* (4.64 ± 0.21%), *Prevotella brevis* (3.83 ± 0.21%), *Prevotella* sp. MA2016 (2.77 ± 0.15%), and *Prevotella bryantii* (2.57 ± 0.44%). For differential abundance comparison analysis at the phylum level, the abundance of *Bacteroidetes* was significantly higher in the rumen of LL cows, while that of *Proteobacteria* was significantly higher in the rumen of HH cows (adjusted *P* < 0.05, Figure S3). At the species level, 15 species, including 11 *Prevotella* sp., one *Succinimonas* sp., one *Selenomonas* sp. and one unclassified *Bacteroidales* exhibited significantly higher abundances in the rumen of HH animals (linear discriminant analysis [LDA] > 2, *P* < 0.05), while 23 species showed significant enrichment in the rumen of LL animals (LDA > 2, *P* < 0.05; Fig. 2a).

For the differential abundance comparison analysis of archaea, the abundance of the most abundant archaeal phylum, *Euryarchaeota* (99.01 ± 0.23%)*,* was significantly higher in the rumen of LL cows (adjusted *P* < 0.01, Figure S4). At the genus level, the abundance of *Methanobrevibacter*, the most abundant archaeal genus (85.44 ± 2.41%), was significantly higher in the rumen of LL cows, while the abundances of other differential genera were all significantly higher in the rumen of HH cows (adjusted *P* < 0.05, Figure S4). At the species level, only the abundance of *Methanobrevibacter millerae* (22.10 ± 2.31%), the most abundant archaeal species, was significantly higher in the rumen of LL cows, while the abundances of the other differential species were all higher in the rumen of HH cows (LDA > 2, *P* < 0.05, Fig. 2b).

### Functional profiles of the rumen microbiome and differential functions between the HH and LL cows

**Fig. 3:**
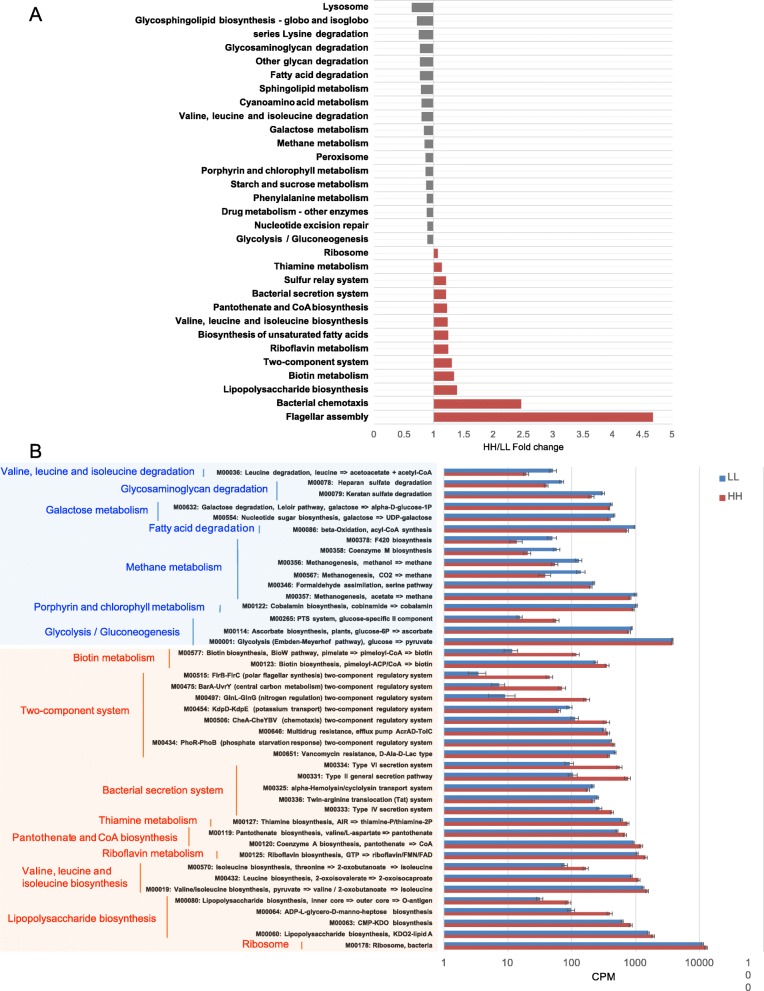
Differential KEGG functions between HH and LL cows. **a** HH/LL fold change of significantly enriched metabolic pathways. **b** Comparison of rumen microbial KEGG modules between HH and LL cows. Significantly different modules in each significantly different level 3 pathway (light orange: higher in HH; light blue: higher in LL) were presented

**Fig. 4:**
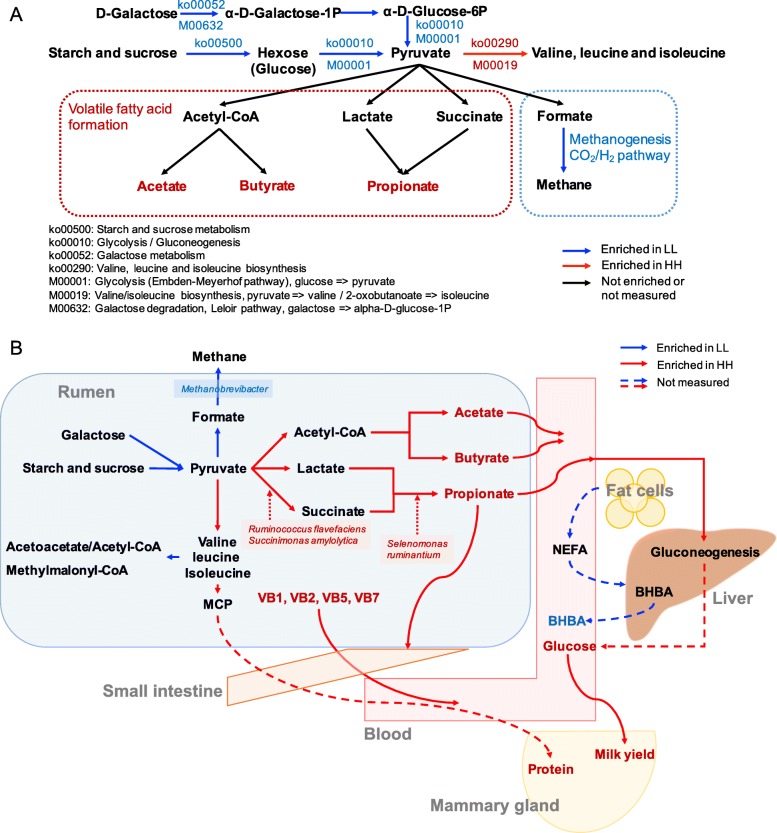
Microbial functions and species involved in carbohydrate metabolism and energy metabolism in the rumen of HH and LL cows. **a** Metabolic pathways involved in VFAs biosynthesis and methanogenesis (CO_2_/H_2_ pathway). **b** Consolidation of results from the rumen microbial taxa, pathways, and rumen volatile fatty acids.

The functions of the rumen microbiome were determined by the Kyoto Encyclopedia of Genes and Genomes (KEGG) profiles and genes encoding CAZymes. For KEGG profiles, 158 endogenous third-level pathways were considered as rumen microbial metabolic pathways (Table S4). These pathways belonged to four first-level categories, including “Metabolism” (72.26 ± 0.46%), “Genetic information processing” (19.08 ± 0.12%), “Environment information processing” (4.42 ± 0.03%), and “Cellular processes” (4.24 ± 0.04%). At the second level, 20 categories were observed, with “Carbohydrate metabolism” (17.33 ± 0.10%), “Amino acid metabolism” (15.96  ± 0.11%), “Nucleotide metabolism” (9.82 ± 0.06%), “Replication and repair” (8.71 ± 0.06%), and “Energy metabolism” (8.07 ± 0.05%) being the most abundant. When the identified KEGG pathways were compared, a total of 13 third-level pathways, including two “Cellular processes” pathways, two “Genetic information processing” pathways, two “Environmental information processing” pathways, and seven “Metabolism” pathways, were significantly enriched in the rumen microbiomes of HH cows, while 18 pathways, including one “Genetic information processing pathway”, two “Cellular processes pathways” and 15 “Metabolism” pathways, were significantly enriched in the rumen of LL animals (LDA > 2 and *P* < 0.05; Fig. 3a). When the KEGG modules involved in the above differential third-level pathways were compared, 24 HH-enriched and 19 LL-enriched modules were identified (Fig. 3b). Regarding carbohydrate metabolism and energy metabolism, only two downstream functions (ko00290 and M00019, converting pyruvate to valine and isoleucine) were enriched in the rumen of HH cows (Fig. 4a). Four pathways and two modules were significantly enriched in the rumen of LL animals (LDA > 2, *P* < 0.05). The four pathways included “Glycolysis” (ko00010), “Starch and sucrose metabolism” (ko00500), “Galactose metabolism” (ko00052), and “Methane metabolism” (ko00680). The two modules were “Glycolysis” (M00001) and “Galactose degradation” (M00632). The downstream function of “Valine, leucine and isoleucine degradation” (ko00280) was also enriched in the rumen of the LL cows (Fig. 4b).

For CAZyme profiles, a total of 313 genes encoding CAZymes were identified (Table S5), including 8 auxiliary activities (AAs), 79 carbohydrate-binding modules (CBMs), 16 carbohydrate esterases (CEs), 115 glycoside hydrolases (GHs), 74 glycosyltransferase (GTs), and 21 polysaccharide lyases (PLs). Among them, genes encoding GT2 (8.64 ± 0.04%) were the most dominant, followed by those encoding CE1 (4.66 ± 0.02%), GT4 (4.34 ± 0.02%), GH2 (4.30 ± 0.02%), and GH3 (4.16 ± 0.02%). Among the genes encoding CAZymes involved in deconstructing carbohydrates (including cellulose, hemicellulose, starch, protein, and lignin), 18 were enriched in the rumen of HH cows (15 GH, 1 CE, 1 PL, and 1 AA), while 34 were enriched in the rumen of LL cows (27 GH, 4 CE, 2 PL, and 1 AA; Figure S5). Among the GTs (involved in carbohydrate synthesis), 11 were enriched in the rumen of HH cows, while two were enriched in the rumen of LL cows. Regarding the CBMs, the noncatalytic CAZymes that are involved in the degradation of complex carbohydrates, three were enriched in the rumen of HH cows, while 19 were enriched in the rumen of LL cows.

### Associations between microbial species and microbial functions

**Fig. 5:**
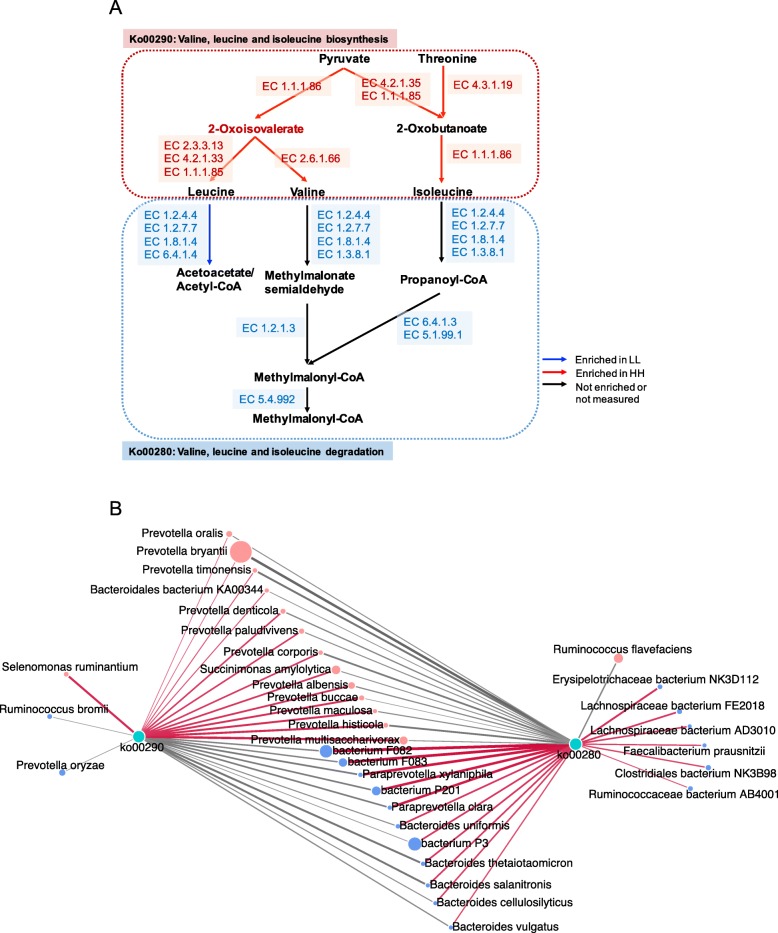
Microbial functions and species involved in branched-chain amino acid (BCAA) metabolism in the rumen of HH and LL cows. **a** BCAAs biosynthesis and degradation pathways. **b** Correlation networks showed associations between significantly different bacterial species and two BCAA pathways. The edge width and color (red: positive, grey: negative) are proportional to the correlation strength. The node size and color (red: significantly enriched in HH; blue: significantly enriched in LL) are proportional to the mean abundance in the respective population. Only strong (Spearman R of > 0.5 or < − 0.5) and significant (*P* < 0.05) correlations were displayed

As protein content is one of the determining measurements of MPY, we further focused on the functions of amino acid metabolism in the rumen microbiome. We found two important pathways involved in branched-chain amino acid (BCAA) metabolism (Fig. 5a), which were “valine, leucine and isoleucine biosynthesis” (ko00290, enriched in the rumen of HH cows) and “valine, leucine and isoleucine degradation” (ko00280, enriched in the rumen of LL cows), and these pathways showed a converse enrichment between the HH and LL groups (LDA > 2, *P* < 0.05; Figure S6). The abundances of genes encoding enzymes involved in these two pathways were also compared, showing that the abundances of genes encoding enzymes involved in BCAA biosynthesis were all significantly enriched in the rumen of HH cows, while the abundances of genes encoding enzymes involved in BCAA degradation were all significantly higher in the rumen of LL cows (adjusted *P* < 0.05; Fig. 5a and Figure S7). A Spearman’s rank correlation network between bacterial species and those two BCAA pathways was then created to explore how rumen bacterial species could affect the microbial BCAA functions. A total of 24 species showed significant relationships with two BCAA pathways (*R* > 0.50 and *P* < 0.05), 13 showing positive relationships with a BCAA biosynthesis pathway (ko00290). Among those 13 positive relationships between bacterial species and ko00290, the strongest (*R* > 0.65 and *P* < 0.05) were detected for five *Prevotella* species, including *P*. *multisaccharivorax*, *P*. *histicola*, *P*. *maculosa*, *P*. *buccae*, and *P*. *albensis* (Fig. 5b).

### Rumen metabolome and serum metabolome

**Fig. 6:**
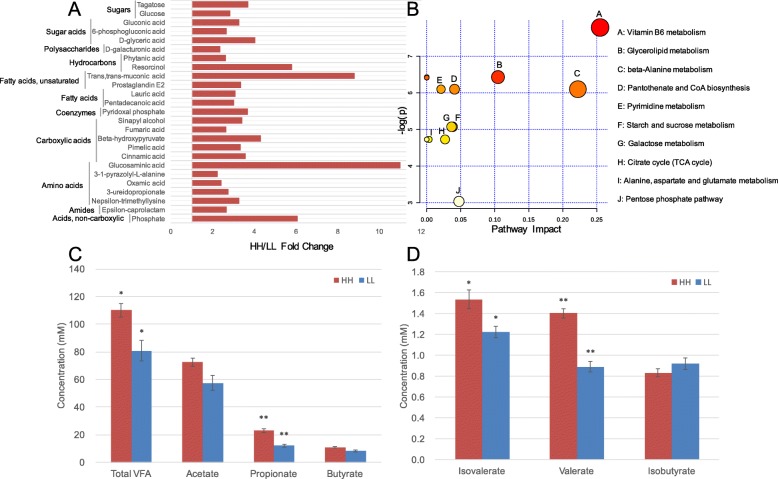
Rumen metabolome of HH and LL cows. **a** HH/LL fold change of significantly different rumen metabolites between HH and LL cows. **b** Pathway enrichment analysis performed using the significantly different rumen metabolites between HH and LL cows. **c** Concentrations of major volatile fatty acids (VFAs) in the rumen of HH and LL cows. **d** Concentrations of minor VFAs in the rumen of HH and LL cows

A total of 263 compounds were identified in the rumen metabolome. After *t* test and variable importance in projection (VIP) filtering for the relative concentrations of rumen metabolites, 25 metabolites were significantly different between the two MPY groups, all of which were significantly higher in the rumen of HH cows (*P* < 0.05, VIP > 1; Fig. 6a). Metabolic pathway analysis (MetPA) based on these 25 significantly different rumen metabolites revealed the enrichment of 10 pathways (Fig. 6b), with “vitamin B6 metabolism”, “glycerolipid metabolism”, and “beta-alanine metabolism” being the significantly different pathways (Benjamini-Hochberg false discovery rate [FDR] < 0.01, pathway impact > 0.1). The rumen metabolome was also used for phenotype (MPY) association analysis, and 126 MPY-associated metabotypes (metabolites that were significantly associated with MPY) were detected (see details in Methods, Table S6). The 126 MPY-metabotypes were used for PERMANOVA analysis; 106 of the MPY-metabotypes (all were MPY-positive metabotypes) were correlated with alterations in the rumen microbiome (adjusted *P* < 0.05; Table S6). These 106 MPY-positive metabotypes were considered as rumen microbiome-responsive metabotypes, which were then found to be significantly associated with 43 microbial modules (*P* < 0.05; Figure S8). In addition to the relative concentrations of ruminal small molecules that were identified by metabolomics, the absolute concentrations of the total volatile fatty acids (VFAs), propionate, valerate, and isovalerate (Fig. 6c, d) were quantified and were significantly higher in the HH cows (*P* < 0.05).

**Fig. 7:**
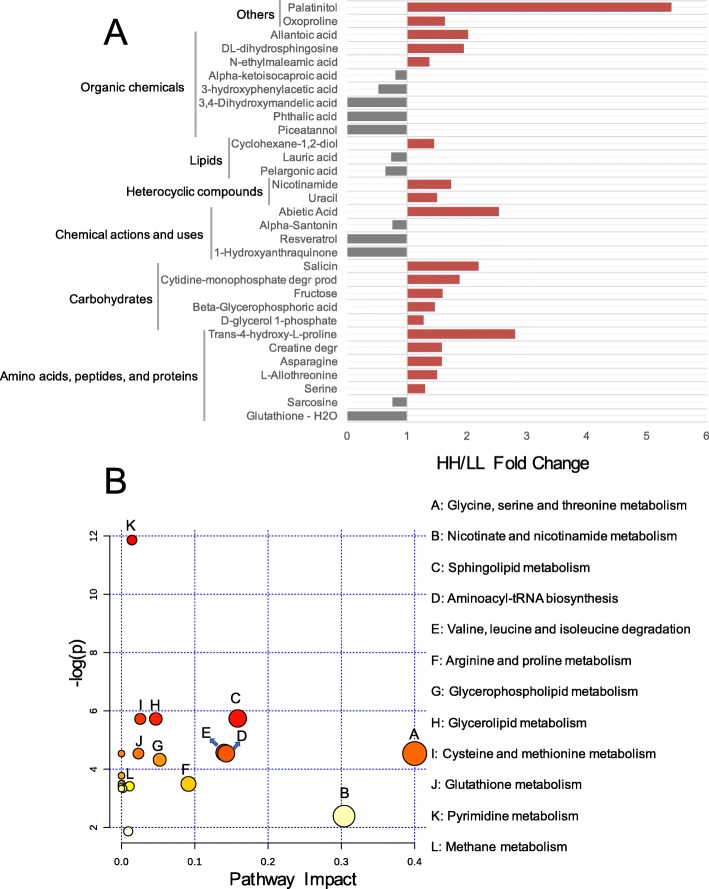
Serum metabolome of HH and LL cows. **a** Comparison of serum metabolome between HH and LL cows visualized using PCA. **b** HH/LL fold change of significantly different serum metabolites between HH and LL cows. **c** Pathway enrichment analysis performed using the significantly different serum metabolites between HH and LL cows

For the serum metabolome, we analyzed the 176 compounds identified in our previous study [6]. The comparison analysis revealed that the relative concentrations of 19 metabolites were significantly higher in the serum of HH cows, and the relative concentrations of 12 metabolites were significantly higher in the serum of LL cows (*P* < 0.05, VIP > 1; Fig. 7a). These 31 significantly different concentrations of metabolites were then used for MetPA analysis, revealing the enrichment of 12 pathways (Fig. 7b), with “glycine, serine, and threonine metabolism”, “nicotinate and nicotinamide metabolism”, and “sphingolipid metabolism”, “aminoacyl-tRNA biosynthesis” and “valine, leucine and isoleucine degradation” being the significantly different pathways (FDR < 0.01, pathway impact > 0.1). The serum metabolome was then identified as MPY-positive metabotypes (21 metabolites) or MPY-negative metabotypes (14 metabolites) using the phenotype (MPY) association analysis as stated above (see details in Methods, Table S7).

To identify whether the MPY-associated metabolites in rumen could be related to those in the serum, we compared the rumen and serum metabolites, including the significantly different metabolites between two MPY groups, MPY-positive metabotypes and MPY-negative metabotypes (Figure S9). A Venn diagram of differential metabolites revealed that a fatty acid, named lauric acid, was shared by the rumen and serum. For the differential metabolite-enriched pathways, three pathways were common in both the rumen and serum of HH cows, including “pyrimidine metabolism”, “glycerolipid metabolism”, and “starch and sucrose metabolism”. The Venn diagram of MPY-associated metabotypes showed that “Arginine and proline metabolism”, “Aminoacyl tRNA biosynthesis”, and “Purine metabolism” were shared by both rumen and serum MPY-positive metabotypes.

### Relationships between the rumen microbiome, rumen metabolome and serum metabolome, and their explainabilities for MPY

**Fig. 8:**
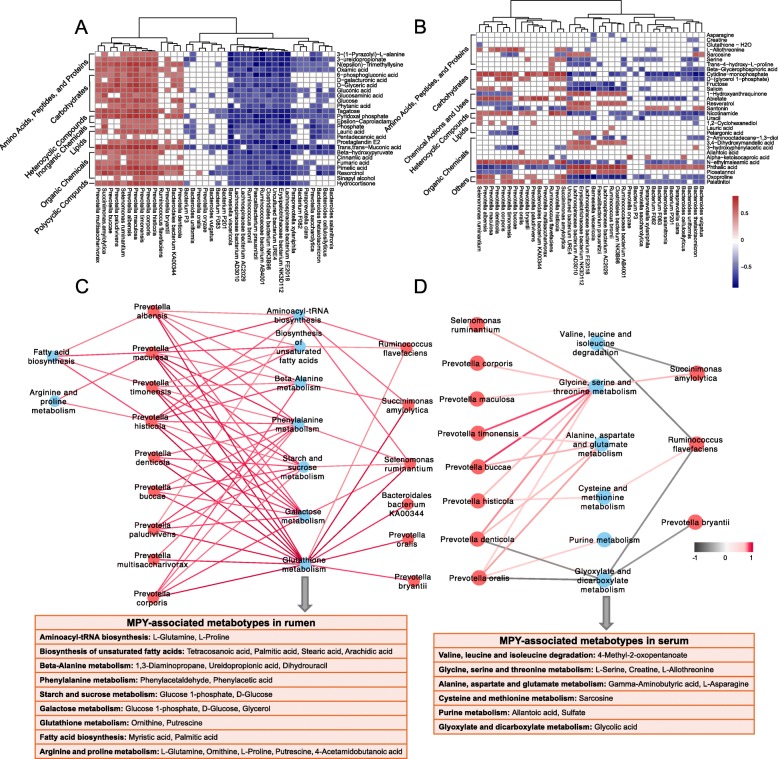
Interactions between rumen metagenome, metabolome, and serum metabolome. **a** Spearman’s rank correlations between rumen microbiota and rumen microbial metabolites. **b** Spearman’s correlations between rumen microbiota and serum metabolites. **c** Spearman’s correlation network showing relationships between rumen microbiota and microbial MPY-associated metabotypes. **d** Spearman’s correlation network showing relationships between rumen microbiota and MPY-associated metabotypes in serum. Only strong correlations (*R* > 0.05 or *R* < − 0.5, *P* < 0.05) were showed in the correlation networks

Spearman’s rank correlations between the rumen microbiota and rumen metabolites were assessed, with the results revealing 65 significant correlations (*R* > 0.50, *P* < 0.05; Fig. 8a). Among the 65 correlations, positive correlations existed between mainly 11 *Prevotella* species (*P. albensis, P. maculosa, P. timonensis, P. histicola, P. denticola, P. buccae, P. paludivivens, P. multisaccharivorax, P. corporis, P. bryantii,* and *P. oralis*) and amino acids, peptides, proteins and organic chemicals (0.50 < *R* < 0.82, *P* < 0.05). Spearman’s rank correlation network showed 22 relationships between the rumen microbiota and MPY-associated metabotypes (Fig. 8c). Among the 22 correlations, nine *Prevotella* species (*P. maculosa, P. histicola, P. denticola, P. buccae, P. paludivivens, P. multisaccharivorax, P. corporis, P. bryantii, P. oralis*) also exihibited correlations with most of the MPY-associated metabotypes, including metabolites involved in glutathione, phenylalanine, starch, sucrose, and galactose metabolism.

To identify the potential rumen microbiome-host metabolic interactions, Spearman’s rank correlations between the rumen microbiota and serum metabolites were performed (Fig. 8b). Fewer relationships existed compared to the relationships identified between the rumen microbiota and rumen metabolites. The relationships between the rumen microbiota and serum MPY-associated metabotypes showed that seven *Prevotella* species were positively correlated with metabotypes involved in the metabolism of several amino acids, including glycine, serine, threonine, alanine, aspartate, glutamate, cysteine, and methionine (Fig. 8d).

**Fig. 9:**
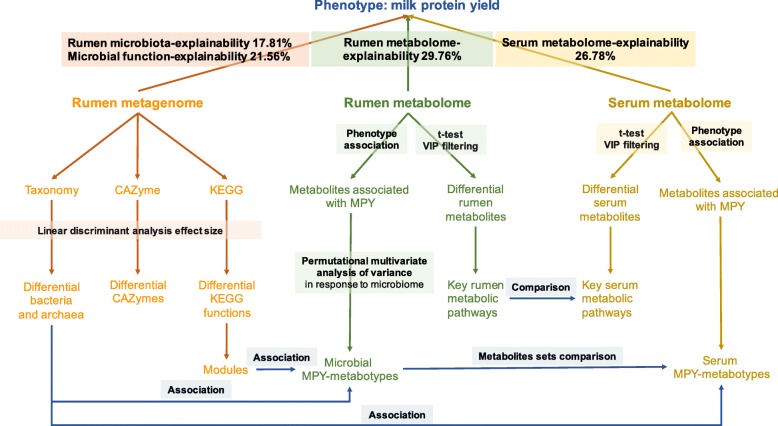
Overview of the workflow. Rumen microbial species and functions (Carbohydrate-active enzymes [CAZymes] and KEGG functions) were compared between two milk protein yield (MPY) groups. Rumen metabolites were separated into two groups that were either positively or negatively correlated with MPY; and then Permutational multivariate analysis of variance (PERMANOVA) was performed based on the microbiome abundance profiles to assess the effect of each metabolites (metabolites with adjusted *P* < 0.05 were considered to associate with rumen microbiota). The rumen metabolome was also separated into two groups that were significantly different between two MPY groups; and the key rumen metabolic pathways were enriched based on the significantly different metabolites. Serum metabolites were separated into two groups that were either positively or negatively correlated with MPY; and were also separated into two groups that were significantly different between MPY groups, which were further enriched for key serum metabolic pathways. The rumen MPY-positive metabotypes and MPY-negative metabotypes were associated with microbiome functional modules. The rumen and serum MPY-positive and MPY-negative metabotypes were clustered into metabolites sets, and were compared. The proportion of variance in MPY explained by the rumen microbial species and functions, rumen metabolome, and serum metabolome (defined as biome-explainability) were estimated

The proportions of variation in MPY due to rumen microbial composition, microbial functions, rumen metabolites, and serum metabolites were estimated using linear mixed effect model (see Methods). The MPY variation explained by the rumen microbial composition, microbial functions, rumen metabolome, and host serum metabolome were 17.81, 21.56, 29.76, and 26.78%, respectively (Fig. 9).

## Discussion

By integrating the rumen metagenome and the rumen and serum metabolomes, we investigated the rumen microbiome-dependent and host metabolome-dependent mechanisms that contribute to MPY and estimated the contributions of the rumen microbial composition, functions, and metabolites to the variations in this trait.

Similar to many previous studies that have assessed rumen microbiomes using metagenomics [17], bacteria were the most abundant rumen microbial kingdom in the rumen of dairy cows and the differences in the rumen microbial features between HH and LL cows were mainly found in bacteria. Consistent with our previous study using 16S rRNA gene amplicon sequencing [7], the bacterial features of the HH and LL cows revealed differences in the relative abundances of taxa at various taxonomic levels. Bacteria are key players in most of the feed biopolymer degradation and fermentation [18], which suggests that the bacteria play more significant roles in contributing to host MPY than other microbial kingdoms. Notably, at the species level, most of the species that showed significantly higher abundances in the HH group belonged to the *Prevotella* genus. This genus utilizes starch and proteins to produce succinate and acetate, and is one of the most abundant core genera in the rumen of dairy cows [19]. The *Prevotella* species, along with *Succinimonas amilolytica* which were over 6-fold enrichment in the rumen of HH cows and act as a succinate-producing bacteria in the bovine rumen [20], showed positive relationships with VFAs concentrations (Figure S10), suggesting their essential roles in VFAs biosynthesis. Additionally, the higher abundances of two succinate-producing and propionate-producing bacteria (*Ruminococcus flavefaciens* and *Selenomonas ruminantium*) in the rumen of the HH cows indicate that these two species might be the main contributors to the higher propionate concentrations in the rumen of the HH cows. Regarding archaea, the higher relative abundances of genus-level *Methanobrevibacter* and species-level *M. millerae* in the rumen of LL animals suggest that the LL cows may produce more methane, leading to less-efficient milk production [11, 17]. In addition to bacteria and archaea, the metagenome has allowed us to identify the rumen microbiome at multi-kingdom levels, including the eukaryote and virus levels. Although we did not focus on eukaryotes or viruses in the current study, their interactions with bacteria could also be a factor affecting host milking traits, which may warrant further studies in the future.

As reported in many other studies [9, 12, 21], the functions of the rumen microbiome are more conserved than the taxonomic composition between two groups of animals. Interestingly, KEGG functions on carbohydrate degradation were enriched in the rumen of LL cows, including “galactose degradation”, “starch and sucrose metabolism”, and the downstream pathway of “glycolysis” that converts glucose to pyruvate, indicating that more hydrolytic products and pyruvate might be generated by the LL microbiome due to the higher ability to degrade carbohydrates. The enrichment of genes encoding CAZymes involved in deconstructing carbohydrates (GH, CE, PL, AA, and CBM) in the rumen microbiomes of LL cows provides further evidence that the LL cows were more capable of degrading complex substrates. However, comparison of downstream pyruvate metabolism pathways and genes encoding relevant enzymes revealed an enrichment of genes involved in methanogenesis in the LL rumen microbiome, together with a higher relative abundance of EC 2.8.4.1, a methyl coenzyme M reductase gene that catalyze the release of methane in the final step of methanogenesis [22]. These results suggest a reduced feed energy in the form of VFAs during microbial fermentation in the LL microbiome [11, 23]. Altogether, although more pyruvate might be supplied by the LL microbiome due to more substrate degradation, the utilization of pyruvate to generate VFAs may not be efficient compared with that of the HH rumen microbiome because the hydrolytic products were ultimately converted to methane (Fig. 4). In contrast to the LL cows, the higher abundances of genes encoding CAZymes that are involved in carbohydrate synthesis (GTs) and the higher concentrations of major VFAs in the rumen of HH animals indicated that the rumen microbiomes of HH cows might be more capable and more efficient in using hydrolytic products to generate VFAs, and therefore provide more energy for lactogenesis in host cows (Fig. 4b). As feed-efficient animals are commonly considered to produce more VFAs and less methane [12, 17, 24], the higher VFAs and lower methanogenic functions in the rumen of HH cows suggest that HH cows may be more feed-efficient than LL cows. Future studies to measure feed efficiency and methane emission are needed to validate our speculations.

In addition to carbohydrates, studies have reported that functions regarding nitrogen metabolism contribute to differential feed efficiency in beef cattle [12] and dairy cows [17]. We compared our results with a dairy study that reported that 13 nitrogen metabolism pathways were enriched in inefficient cows [17] and found that three of the 13 pathways, including “valine, leucine, and isoleucine degradation”, “lysine degradation”, and “phenylalanine metabolism”, were significantly enriched in the LL cow microbiome. Additionally, the BCAAs, including valine, leucine, and isoleucine, are important contributors to microbial protein synthesis [25], with the ruminal microbial protein produced by rumen microbes fulfilling up to 90% of amino acids that arrived to the small intestine [26]. The enrichment of the BCAA biosynthesis functions in the rumen of the HH cows suggest that more microbial protein, which acts as a precursor for the synthesis of milk protein in the mammary gland, might be synthesized in the rumen of the HH cows. Moreover, our results revealed that most species showing positive relationships with BCAA biosynthesis pathway belonged to the *Prevotella* genus*,* suggesting the role of *Prevotella* species in BCAA biosynthesis, which has not been reported previously. Future studies to detect the active microbial functions and taxa, along with culture-based studies are required to confirm the function of those *Prevotella* species in BCAA biosynthesis.

Another important function identified in the current study was vitamin B metabolism. In dairy cows, the vitamin B group is synthesized by the bacteria in the rumen and functions as enzyme cofactors or precursors for cofactors [27]. The vitamin B group is involved in several essential metabolic processes, including fatty acid synthesis, BCAA catabolism, and gluconeogenesis [28]. For example, biotin acts as a cofactor responsible for carbon dioxide transfer in carboxylases [29]; riboflavin functions as cofactors in flavoprotein enzyme reactions, including succinate dehydrogenase and the oxidation of pyruvate [30]; and pantothenate is involved in the synthesis of CoA, which is important for energy metabolism for pyruvate to enter the tricarboxylic acid (TCA) cycle as acetyl-CoA [31]. In our study, functions of the vitamin B complex, including “Biotin metabolism”, “riboflavin metabolism”, “pantothenate and CoA biosynthesis”, and “thiamine metabolism” pathways, were more abundant in the HH rumen microbiome (Figure S5C). Numerous recent studies have reported that supplementation with vitamin B compounds could increase milk yield and/or component yield in dairy cows [28, 32], indicating that a higher vitamin B level is needed for high milk yield and/or component yield production. Thus, it is likely that the rumen microorganism of the HH cows can produce higher amounts of vitamin B, which could contribute to the high MPY. Additionally, there were two other pathways that were significantly enriched in the HH rumen microbiome: “bacterial chemotaxis” (2.5-fold) and “flagella assembly” (4.6-fold). By chemotaxis functions, microbes sense chemical gradients and move towards their favorable nutritional conditions [33], which causes changes in the behavior of microbes such as speed of rotation of flagella and flagella assembly [34]. Although the way in which these pathways affect the overall functions of the rumen microbiome are unclear, we speculate that the microbes in HH cows may be more capable of sensing and moving towards their favorable nutrients than those in LL cows. Future detection of vitamin B production as well as microbial flagellin in the rumen metabolome could provide a better understanding of the contribution of these functions to MPY.

As the outcome of microbiome compositional and functional differences, differences in rumen metabolomes between the two groups were found in this study. Our metagenomics functional-level results revealed that the HH microbiome had less diverse functions but higher concentrations of VFAs than the LL microbiome. In addition to the VFAs, the relative concentrations of several small-molecule metabolites were over 2-fold higher in the rumen of the HH cows. These small-molecule metabolites were mainly carbohydrates and carbohydrate conjugates. For example, the concentrations of glucosaminic acid and phosphate were more than 6-fold higher in the rumen of the HH cows. These conjugate acids involve in the microbial pentose phosphate pathway, suggesting potentially more oxidation in the rumen microbiome and subsequently more energy supply in the HH animals [35]. The higher relative concentrations of these carbohydrates provide evidence to support our previous findings that more metabolic energy was provided to mammary glands of the HH cows via bloodstream [6]. The gas chromatography-based method used in this study separates compounds based on their volatility and polarity, and is one of the best techniques to detect the volatile compounds. Although we detected some non-volatile small molecules after derivatization, other non-volatile compounds with large molecular mass might be under-investigated [36]. More efforts using alternative methods, such as liquid chromatography-based methods, are required to further identify the whole rumen microbial metabolome and to explain the microbial metabolism variation between the HH and LL animals.

In ruminants, the relationship between rumen microbial taxa and the rumen metabolome has been reported using goat as a ruminant model [37]. However, whether and how the rumen metagenome could interact with the microbial metabolome remains unknown. In the current study, we identified the associations between rumen metagenome and rumen microbial metabolome and found that MPY-associated metabotypes were positively correlated with specific microbial taxa, mostly *Prevotella* species. Our results also revealed that rumen MPY-associated metabotypes interacted with 43 microbial KEGG modules as well. Overall, the interactions between microbial taxa and functions with microbial metabolites suggest that the *Prevotella* species may be crucial contributors to microbial metabolites including amino acids and carbohydrates involved in glutathione, phenylalanine, starch, sucrose, and galactose metabolisms. The relationships between the rumen microbial taxa, functions, and metabolome provide new insights into the functional roles of the rumen microbiome in producing small molecule metabolites and contributing to host traits.

Recent papers have reported that the host, together with the rumen microbiome, affect host traits, including methane production [14], feed efficiency [15], and milking traits [38] in dairy cows. The findings from our current study suggest that the rumen metagenome, rumen metabolome, and host serum metabolome all influenced the host MPY [6] similar to the effects on the traits mentioned above. In our study, the associations between the rumen microbiome and serum metabolome suggest that the rumen microbiome potentially interacts with host metabolism. Notably, *Prevotella* species may affect host amino acid metabolism, including glycine, serine, threonine, alanine, aspartate, glutamate, cysteine, and methionine. We then estimated the proportions of variation in MPY due to rumen microbial composition, microbial functions, microbial metabolites, and host metabolites. Inspired by the concept of biome-explainability which was defined as the variance in host phenotype explained by the microbiome in a human study [39], we defined such proportion of variation as “omics-explainability” in our study. In animals, this concept was first proposed by Difford et al. in dairy cows and was defined as “microbiability”, estimated by quantifying the cumulative effects of microbial abundance on phenotypes [14]. Such a concept has also been applied in pigs [40] and chickens [41]. In a recent dairy study, the authors found that the cumulative effect of bacteria and archaea identified by 16S rRNA gene amplicon sequencing explained 13% of the individual variation in methane production [14]. Using metagenomics, we found that the cumulative effect of rumen microbial composition (17.81%) and functions (21.56%) on the variation in MPY was higher than that reported for methane production. This difference may be due to the more comprehensive information on the rumen microbiota characterized by metagenomics compared with amplicon sequencing, since multi-kingdom levels including not only bacteria and archaea but also eukaryotes and viruses can be characterized by metagenomic sequencing. In addition to the microbiability, the omics-explainability of the metabolome has not yet been reported. The calculation of omics-explainability of the rumen and serum metabolome in our study suggests that the metabolism of the rumen and host potentially make greater contributions to MPY compared with the contributions of the rumen microbiome and functions. Although the rumen microbial taxonomy and functions has been considered to play roles in efficiency [12, 17] and milking traits [7], our findings suggest that the rumen microbial metabolites should be routinely considered in addition to the microbiome in future studies aimed at improving host efficiency and milking traits. Additionally, by calculating omics-explainability, researchers have proposed that the characteristics of the rumen microbiota could be used as new selection criteria to manipulate the host phenotype in dairy cows, such as methane emission, in addition to genome-wide selection in dairy breeding [14]. Our omics-explainability results further suggest that even better prediction of milking traits may be obtained by using rumen metabolites, and the prediction can be related to any other trait associated with rumen function and metabolism. Further study to detect the prediction accuracy of various omics data for milking traits, compared to models that use only host genetic data will provide more evidence for this potentially new selection criteria.

Although the factors affecting the MPY of dairy cows including feed, management, age, and lactation stage were largely controlled in our study, we found that the variation in host MPY were also attributed to the variations in rumen microorganism and its metabolites, as well as the utilization and absorption of metabolites by the host. In addition to the factors mentioned above, this milking trait could also be attributed to variations in feed intake and genetics. The differential methanogen and methanogenesis functions, along with VFA biosynthesis functions and VFA concentrations, indicate differential methane production and feed efficiency, which need to be further confirmed. Furthermore, recent amplicon sequencing-based studies have reported that ruminant genetics influenced not only phenotypes but also the rumen microbiota, and the heritable microbial taxa were associated with feed efficiency [15, 42] and methane emission [14]. Due to a lack of knowledge regarding the heritability of microbial functions and relevant output metabolites, as well as their contribution to milking traits, future studies are required to assess the heritability of those functional and metabolic elements. Such information will provide evidence highlighting the possibility of manipulating rumen microbial functions and metabolites through genetic selection.

## Conclusion

Our study identified the rumen microbial taxonomic features, functions, metabolites together with their interactions with host metabolism that contribute to host MPY. Cows with higher MPY had lower abundances of archaeal species and functions in methanogenesis, leading to higher functions and enzymes involved in carbohydrate synthesis. Several *Prevotella* species were enriched in the HH cows and were associated with BCAA biosynthesis functions, ruminal amino acids, and serum amino acids, fulfilling the demand for rumen microbial proteins that are utilized by hosts for milk protein biosynthesis. The microorganisms in the rumen of HH cows serve as stronger vitamin B producers, meeting the requirement for higher milking performances. As the outcome of the microbiome structural and functional differences, the levels of the small molecular metabolites (mainly amino acids, carboxylic acids, and fatty acids) and end products (VFAs) of the HH microbiome were higher, contributing to differences in metabolites absorbed and transported by the host. Altogether, omics-explainability analysis revealed that the rumen microbial metabolites and serum metabolites made greater contributions to MPY than rumen microbial composition and functions. The microbiome-dependent and host-dependent mechanisms contributing to MPY provide insights into strategies for altering the rumen microbiota for higher milk quality and production through either feeding management or genetic selection.

## Methods

### Animals, sampling, and physiological parameters measurement

Based on previous milking trait measurements [7], 10 highest MPY cows (cows with high milk yield and milk protein content; HH) and 10 lowest-MPY cows (cows with low milk yield and milk protein content; LL) were selected from the cohort of 374 healthy mid-lactation Holstein dairy cows hosed at a commercial dairy farm. Animals received the same diet with a concentrate-to-forage ratio of 57:43 (dry matter basis) [6]. Rumen contents were sampled using oral stomach tubes and were used to measure VFAs [7]. Blood was sampled to measure chemical parameters in serum [6].

### DNA extraction, metagenome sequencing, and metagenomics data processing

Total genomic DNA was extracted from rumen contents using the repeat bead-beating plus column method [43]. The quality and quantity of DNA were measured using a NanoDrop 2000 spectrophotometer (NanoDrop Technologies, Wilmington, DE, USA). After quantity measurement of DNA samples, four samples (three from HH and one from LL) were discarded due to low DNA quantity. Power calculations revealed that our sample size enables 87.5% power and a type I error of 5%, based on *t* test of MPY. Construction of metagenome libraries was performed using TrueSeq DNA PCR-Free Library Prep Kits (Illumina, San Diego, CA, USA). Metagenome libraries sequencing was performed on an Illumina Hiseq 3000 platform (150 bp paired-end sequencing, 500 pb inserts) at Majorbio Bioinformatics Technology Co. Ltd. (Shanghai, China).

The quality control of each dataset was performed using Sickle (version 1.33, https://github.com/najoshi/sickle) to trim the 3’-end of reads and 5’-end of reads, cut low-quality bases (quality scores < 20), and remove short reads (< 50 bp) and “N” records. The reads were aligned to the bovine genome (bosTau8 3.7, DOI: 10.18129/B9.bioc.BSgenome.Btaurus.UCSC.bosTau8) using BWA (http://bio-bwa.sourceforge.net) to filter out host DNA [44]. The filtered reads were de novo assembled for each sample using Megahit (https://github.com/voutcn/megahit) [45]. MetaGene (http://metagene.cb.k.u-tokyo.ac.jp/) was used to predict open reading frames (ORFs) from the assembled contigs with the length > 300 bp [46]. Assembled contigs were then pooled and non-redundancies were constructed based on the identical contigs using CD-HIT with 95% identity (http://www.bioinformatics.org/cd-hit/) [47]. Original sequences were mapped to predicted genes to estimate the abundances using SOAPaligner (http://soap.genomics.org.cn/) [48].

### Taxonomic and functional annotation from rumen metagenomes

Taxonomic assessment of rumen microbiota was performed using DIAMOND (http://ab.inf.uni-tuebingen.de/software/diamond) [49] against the RefSeq database (http://www.ncbi.nlm.nih.gov/RefSeq/) [50]. Taxonomic profiles were conducted at domain, phylum, genus and species levels, with relative abundances calculated. The PCoA based on Bray-Curtis dissimilarity matrices at species level was performed. Microbial taxa with a relative abundance > 0.1% in at least 50% of cows within each group were used for downstream analysis.

Contigs were annotated using DIAMOND against the KEGG database (http://www.genome.jp/kegg/) with an E value of 1e-5 [51]. The CAZy annotation was performed using USEARCH (http://www.drive5.com/usearch/) [52]. Abundances of KEGG Orthology (KO), pathway, KEGG enzyme, Module, and CAZymes were normalized into counts per million reads (cpm) for downstream analysis. The KEGG modules, pathways, KEGG enzymes, and CAZymes with cpm > 5 in at least 50 % of animals within each group were used for the downstream analysis.

### Analysis of rumen and serum metabolome

The rumen metabolome [53] and serum metabolome [6] were analyzed using gas chromatography (Agilent Technologies, Santa Clara, CA, USA) combined with Pegasus HT time-of-flight/ mass spectrometry (GC-TOF-MS, LECO Corporation, St. Joseph, MI, USA). Chroma TOF 4.3X software (LECO Corporation, St. Joseph, MI, USA) and LECO-Fiehn Rtx5 database [54] were used for raw peaks exacting, data baseline filtering and calibration of the baseline, peak alignment, deconvolution analysis, peak identification, and integration of the peak area. Both of mass spectrum match and retention index match were considered in metabolites identification. Rumen and serum metabolite peaks that were present in < 50% of samples or with relative standard deviation>30% or with similarity value < 200 were removed [55]. The unidentified peaks were also removed from the downstream analysis. In total, 263 rumen metabolites and 177 serum metabolites were identified and were transformed to have a zero mean and a unit variance for downstream analysis.

The online platform, MetaboAnalyst 4.0 (https://www.metaboanalyst.ca/MetaboAnalyst/faces/home.xhtml) [56], was used for the MetPA based on targeted metabolites using the library of Bos Taurus (cow) [57]. Metabolite set enrichment analysis (MESA) was performed using MetaboAnalyst 4.0, based on the pathway-associated metabolite sets library [58]. The metabolite datasets in serum and rumen were compared between the two MPY groups and visualized using heat maps (“pheatmap” package in R, https://www.r-project.org) [59].

### Calculation of omics-explainability

Species-level microbial relative abundances, KOs, rumen metabolites, and serum metabolites were normalized to have a zero mean and a unit variance and then were used to construct the matrix **M**, **K**, **R**, and **S**, respectively [14]. The LMM utilized to estimate the variances of four omics was calculated as follows:

1 $$ {y}_{ijk}=\mu +{p}_j+{d}_k+{m}_i+{e}_{ijk} $$

where *y*_*ijk*_is the phenotype MPY (kg/day); *μ* is the model intercept; *p*_*j*_ is the parity fixed effect; *d*_*k*_ is the days-in-milk fixed effect; *m*_*i*_ is the rumen microbial random effect for the *i*th $\mathrm{animal}\sim \mathrm{NID}\ \left(0,\mathbf{M}{\sigma}_m^2\right)$, where ${\sigma}_m^2$ is the rumen microbial variance and **M** is the microbial relationship matrix; and *e*_*ijk*_ is the residual effects. The LMM utilized to estimate the KO variance was similar to Eq. (1), except the random effect of *k*_*i*_, which is the random effect of the KOs for the $i\mathrm{th}\ \mathrm{animal}\sim \mathrm{NID}\ \left(0,\mathbf{K}{\sigma}_k^2\right)$, where ${\sigma}_k^2$ is the rumen microbial variance and **K** is the rumen functional relationship matrix. The LMM utilized to estimate the rumen metabolic variance was similar to Eq. (1), except the random effect of *r*_*i*_, which is the random effect of the rumen metabolites for the $i\mathrm{th}\ \mathrm{animal}\sim \mathrm{NID}\ \left(0,\mathbf{R}{\sigma}_r^2\right)$, where ${\sigma}_r^2$ is the rumen microbial variance and **R** is the rumen functional relationship matrix. The LMM utilized to estimate the serum metabolic variance was similar to Eq. (1), except the random effect of *s*_*i*_, which is the random effect of the serum metabolites for the $i\mathrm{th}\ \mathrm{animal}\sim \mathrm{NID}\ \left(0,\mathbf{S}{\sigma}_s^2\right)$, where ${\sigma}_s^2$ is the rumen microbial variance and **S** is the rumen functional relationship matrix. The MPY variance that explained by the rumen microbial variance, functional variance, rumen metabolic variance, and serum metabolic variance were estimated as $\frac{\sigma_m^2}{\sigma_p^2}$, $\frac{\sigma_k^2}{\sigma_p^2}$, $\frac{\sigma_r^2}{\sigma_p^2}$, and $\frac{\sigma_s^2}{\sigma_p^2}$, respectively, where ${\sigma}_p^2$ is the phenotypic (MPY) variance.

The LMM was performed using the “lme4” package in R (https://www.r-project.org) [60]. The *P* values of the omics-explainability estimations were calculated by using the likelihood ratio tests on the random effect (*P* < 0.05). The random effect will be accepted when the likelihood ratio test reveal that fitting the random effect of omics data being significantly better than the null hypothesis (the variance of the random effect is 0).

### Correlation analysis

Correlation analysis between rumen metabolites, serum metabolites and MPY was performed using Spearman’s rank correlation to identify the MPY-associated metabotypes (“MPY- associated metabotypes”), with *P* value (Spearman’s rank correlation coefficient) < 0.05 being considered as significantly MPY-associated metabotypes. To identify the associations between microbial composition and each MPY-associated metabotypes covariate, we performed the permutation multivariate analysis of variance (PERMANOVA) on the microbial abundance profiles [61] using microbial Bray–Curtis distance in R “vegan” package (https://www.r-project.org) [62]. Rumen metabolites with FDR adjusted *P* < 0.05 were considered to be associated with rumen microbiota and were subsequently used for correlation analysis with KEGG modules.

All correlation analyses were performed using Spearman’s rank correlation, and *P* value < 0.05 was considered as significant. Correlation network was visualized by Cytoscape (Version 3.2.1, http://www.cytoscape.org). The correlation heat map was generated using the R program “pheatmap” package (https://www.r-project.org) [59].

## Statistical analysis

The statistical analyses were performed using the “stats” package in R (https://www.r-project.org) [63]. Milking traits, serum biochemical parameters, and rumen VFAs concentrations were compared using *t* test. Rumen microbial domains, phyla, and genera were compared using Wilcoxon rank-sum test, with the FDR adjusted *P* value < 0.05 being considered as significantly different. Rumen microbial species were compared using linear discriminant analysis effect size (LEfSe) [64], and significant differences were examined by a LDA score > 2 and *P* value < 0.05. The abundances of microbial metabolic pathways, modules, KEGG enzymes, and CAZymes were compared between two groups using LEfSe, and significant differences were considered by an LDA score > 2 and *P* value < 0.05.

The MetaboAnalyst 4.0 was used to perform the multivariate analysis and statistical analysis for metabolome data. The PCA, partial least squares discriminant analysis (PLS-DA), and *t* test were performed between the two MPY groups, with the FDR adjusted *P* value < 0.05 and the VIP > 1 being considered as significantly different metabolites.

## Supplementary information

**Additional file 1: Table S1.** Physiological parameters of HH and LL cows.

**Additional file 2: Table S2.** Summary of sequence data generated from rumen samples of 7 HH and 9 LL cows.

**Additional file 3: Figure S1.** Profiles of rumen microbial composition of dairy cows. (A) Rumen microbial composition based on the domain-level taxonomy. (B) Bacterial composition based on the phylum-, family-, and genus-level taxonomy. (C) Archaeal composition based on the phylum-, family-, and genus-level taxonomy. (D) Eukaryotic composition based on the kingdom- and phylum-level taxonomy.

**Additional file 4: Table S3.** PERMANOVA (permutational multivariate analysis of variance) of three microbial domains between HH and LL samples.

**Additional file 5: Figure S2.** Microbial compositional profiles of (A) Eukaryota and (B) Viruses of the HH and LL rumen samples visualized using principal-coordinate analysis (PCoA). The first two PCoAs were plotted, and calculated based on the Bray-Curtis dissimilarity matrices at species level.

**Additional file 6: Figure S3.** Comparison of bacterial phyla and genera. Bacterial phyla (A) and genera (B) were tested by Wilcoxon rank-sum test, *P<0.05, **P<0.01.

**Additional file 7: Figure S4.** Comparison of archaeal phyla and genera. Archaeal phyla (A) and genera (B) were tested by Wilcoxon rank-sum test, *P<0.05, **P<0.01.

**Additional file 8: Table S4.** Composition of metabolic pathways based on the first-level and second-level functions in the KEGG.

**Additional file 9: Table S5.** Composition of CAZymes based on the class-level and family-level enzymes.

**Additional file 10: Figure S5.** Differential CAZyme functions between HH and LL cows. (A) Significantly different Glycoside Hydrolases (GHs), Carbohydrate Esterases (CEs), Polysaccharide Lyases (PLs), and Auxiliary Activities (AAs) between the rumen of HH and LL cows. (B) Significantly different GlycosylTransferases (GTs) between the rumen of HH and LL cows. (C) Significantly different Carbohydrate-Binding Modules (CBMs) between the rumen of HH and LL cows. Significantly different CAZymes were tested by Linear discriminant analysis effect size (LEfSe) analysis with linear discriminant analysis (LDA) score of > 2 and *P* value of < 0.05.

**Additional file 11: Figure S6.** HH/LL fold change shows differences in level-3 microbial pathways between HH and LL cows. (A) Amino acid metabolism. (B) Carbohydrate metabolism. (C) Metabolism of cofactors and vitamins. (D) Energy metabolism. Significant different pathways were tested by Linear discriminant analysis effect size (LEfSe) analysis with linear discriminant analysis (LDA) score of > 2 and *P* value of < 0.05.

**Additional file 12: Figure S7.** Comparison of significantly enriched ECs involved in branched chain amino acid biosynthesis (A) and degradation (B). Significantly different pathways were tested by Wilcoxon rank-sum test with adjusted *P* value of < 0.05.

**Additional file 13: Table S6**. Phenotype (MPY)-associated metabolites in rumen.

**Additional file 14: Figure S8.** Association heat map between MPY-positive associated metabotypes and microbiome functional modules.

**Additional file 15: Table S7**. Phenotype (MPY)-associated metabolites in serum.

**Additional file 16: Figure S9.** Comparison of MPY-associated metabolites, metabolites-enriched pathways, and metabolites sets between rumen and serum. (A) The Venn diagram shows significantly different metabolites in rumen and serum between different MPY groups. (B) The Venn diagram shows key pathways (enriched based on the significantly different metabolites). (C) Venn diagram shows MPY-positive and MPY-negative metabolites sets in rumen and serum. Heat maps display the Z score-transformed abundance of each metabolites sets in each sample.

**Additional file 17: Figure S10.** Associations between significantly enriched bacterial species and metabolites, metabolic pathways, and phenotypes. Only significant correlations (*P* < 0.05) were presented in the correlation heat map. Red: significantly positive correlations, blue: significantly negative correlations, white: no significant correlation.

## Data Availability

The rumen metagenome sequences were deposited into NCBI Sequence Read Archive (SRA) under the accession number PRJNA526070 (https://www.ncbi.nlm.nih.gov/bioproject/PRJNA526070).
