# Supplementary material for: Multi-omics reveals that the rumen microbiome and its metabolome together with the host metabolome contribute to individualized dairy cow performance
Source: Microbiome. 2020 May 12;8:64. doi: 10.1186/s40168-020-00819-8 (PMC7218573; doi:10.1186/s40168-020-00819-8)
Supplement: Supplementary file 2 — Additional file 1: Table S1. Physiological parameters of HH and LL cows. [file 40168_2020_819_MOESM1_ESM.docx]

**Table S1 Physiological parameters of HH and LL cows.**

|  | Mean | |  | |  |
| --- | --- | --- | --- | --- | --- |
| Items | HH (n=7) | LL (n=9) | SEM | | *P* |
| Performance  Milk yield (kg/d) | 37.20 | 25.87 | 1.73 | < 0.01 | |
| Milk protein (%) | 3.31 | 2.72 | 0.09 | < 0.01 | |
| Milk fat (%) | 2.76 | 3.98 | 0.36 | 0.10 | |
| Lactose (%) | 4.98 | 4.98 | 0.05 | 0.96 | |
| Milk protein yield (kg/d) | 1.23 | 0.70 | 0.07 | < 0.01 | |
| Parity | 2.14 | 2.78 | 0.16 | 0.21 | |
| DIM (d) | 170.71 | 146.56 | 8.26 | 0.15 | |
| Serum biochemical parameters |  |  |  |  | |
| Albumin, g/L | 38.96 | 39.65 | 1.00 | 0.74 | |
| Blood urea nitrogen, mmol/L | 4.88 | 3.99 | 0.35 | 0.21 | |
| Creatinine, mmol/L | 69.82 | 60.50 | 3.83 | 0.24 | |
| β-hydroxybutyrate, mmol/L | 0.41 | 0.64 | 0.06 | 0.05 | |
| Glucose, mmol/L | 4.47 | 3.97 | 0.15 | 0.10 | |
| Glutamic-oxalacetic transaminease, U/L | 8.00 | 9.65 | 0.53 | 0.13 | |
| Glutamic-pyruvic transaminase, U/L | 5.43 | 4.64 | 0.50 | 0.45 | |
| Glutathione peroxidase, U/mL | 130.64 | 129.33 | 5.06 | 0.90 | |
| Malonaldehyde, nmol/mL | 3.85 | 3.08 | 0.28 | 0.18 | |
| NEFA, mmol/L | 0.24 | 0.22 | 0.02 | 0.59 | |
| Superoxide dismutase, U/mL | 139.99 | 135.25 | 3.13 | 0.47 | |
| Total antioxidant capability, U/mL | 3.81 | 3.68 | 0.19 | 0.76 | |
| Total bilirubin, mmol/L | 5.78 | 3.82 | 0.43 | 0.02 | |
| Total cholesterol, mmol/L | 6.32 | 5.44 | 0.33 | 0.19 | |
| Triglyceride, mmol/L | 0.29 | 0.31 | 0.02 | 0.64 | |
| Total protein, g/L | 60.97 | 67.59 | 2.19 | 0.14 | |

SEM, stand error of the mean. *P* Values between HH and LL cows were calculated using t test.
