# Supplementary material for: Multi-omics reveals that the rumen microbiome and its metabolome together with the host metabolome contribute to individualized dairy cow performance
Source: Microbiome. 2020 May 12;8:64. doi: 10.1186/s40168-020-00819-8 (PMC7218573; doi:10.1186/s40168-020-00819-8)
Supplement: Supplementary file 3 — Additional file 2: Table S2. Summary of sequence data generated from rumen samples of 7 HH and 9 LL cows. [file 40168_2020_819_MOESM2_ESM.docx]

**Table S2 Summary of sequence data generated from rumen samples of 7 HH and 9 LL cows.**

| Sample | Raw reads | Reads after QC | Clean reads | Contigs | N50(bp) | ORFs |
| --- | --- | --- | --- | --- | --- | --- |
| 2_16 | 61,701,178 | 59,659,066 | 59,522,697 | 684,375 | 717 | 861,841 |
| 2_31 | 59,141,244 | 57,154,663 | 57,024,019 | 697,239 | 846 | 952,269 |
| 2_45 | 69,740,990 | 67,779,480 | 67,624,550 | 910,704 | 719 | 1,157,605 |
| 4_58 | 73,330,896 | 70,983,070 | 70,820,817 | 889,285 | 743 | 1,117,530 |
| 4_85 | 60,413,100 | 58,117,909 | 57,985,063 | 667,335 | 716 | 862,065 |
| 4_89 | 70,750,268 | 68,860,678 | 68,703,276 | 747,940 | 853 | 998,413 |
| 4_96 | 66,521,710 | 63,957,428 | 63,811,234 | 725,145 | 717 | 929,315 |
| 6_45 | 68,288,890 | 66,610,591 | 66,458,333 | 825,853 | 767 | 1,011,767 |
| 6_86 | 65,282,512 | 63,167,976 | 63,023,587 | 718,121 | 803 | 932,872 |
| 7_42 | 68,362,320 | 66,384,064 | 66,232,323 | 496,290 | 1,154 | 721,588 |
| 7_57 | 73,324,032 | 71,415,432 | 71,252,191 | 861,279 | 811 | 1,142,276 |
| 7_67 | 65,283,008 | 63,392,831 | 63,247,928 | 627,209 | 846 | 833,544 |
| 8_19 | 64,921,186 | 62,484,893 | 62,342,065 | 788,289 | 723 | 1,016,418 |
| 8_33 | 63,081,890 | 60,982,526 | 60,843,132 | 756,088 | 827 | 990,531 |
| 8_36 | 64,633,852 | 62,766,859 | 62,623,387 | 802,509 | 787 | 1,062,413 |
| 8_69 | 74,654,404 | 72,253,976 | 72,088,818 | 899,632 | 679 | 1,133,143 |
| Total | 1,069,431,480 | 1,035,971,442 | 1,033,603,420 | 12,097,293 | 12,708 | 15,723,590 |
| mean | 66,839,468 | 64,748,215 | 64,600,214 | 756,081 | 795 | 982,724 |
| SD | 4,675,960 | 4,673,456 | 4,662,773 | 110,884 | 111 | 124,193 |
| SEM | 1,168,990 | 1,168,364 | 1,165,693 | 27,721 | 28 | 31,048 |
