# Supplementary material for: Multi-omics reveals that the rumen microbiome and its metabolome together with the host metabolome contribute to individualized dairy cow performance
Source: Microbiome. 2020 May 12;8:64. doi: 10.1186/s40168-020-00819-8 (PMC7218573; doi:10.1186/s40168-020-00819-8)
Supplement: Supplementary file 5 — Additional file 4: Table S3. PERMANOVA (permutational multivariate analysis of variance) of three microbial domains between HH and LL samples. [file 40168_2020_819_MOESM4_ESM.docx]

**Table S3 PERMANOVA (permutational multivariate analysis of variance) of three microbial domains between HH and LL samples.**

| Taxonomy | SumsOfSqs | MeanSqs | F. Model | R2 | *P*. adjust |
| --- | --- | --- | --- | --- | --- |
| *Bacteria* | 0.100 | 0.100 | 12.999 | 0.481 | 0.001 |
| *Eukaryota* | 0.386 | 0.386 | 1.823 | 0.115 | 0.174 |
| *Archaea* | 0.876 | 0.876 | 11.328 | 0.447 | 0.002 |
| *Viruses* | 0.581 | 0.581 | 4.256 | 0.233 | 0.064 |
