# Supplementary figures and images for: Multi-omics reveals that the rumen microbiome and its metabolome together with the host metabolome contribute to individualized dairy cow performance
Source: Microbiome. 2020 May 12;8:64. doi: 10.1186/s40168-020-00819-8 (PMC7218573; doi:10.1186/s40168-020-00819-8)

A

PCoA analysis

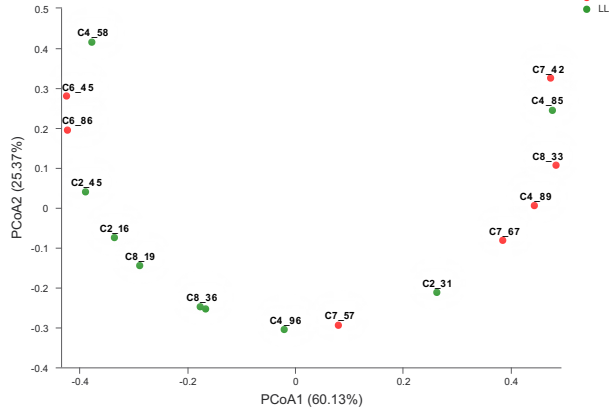

B

PCoA analysis

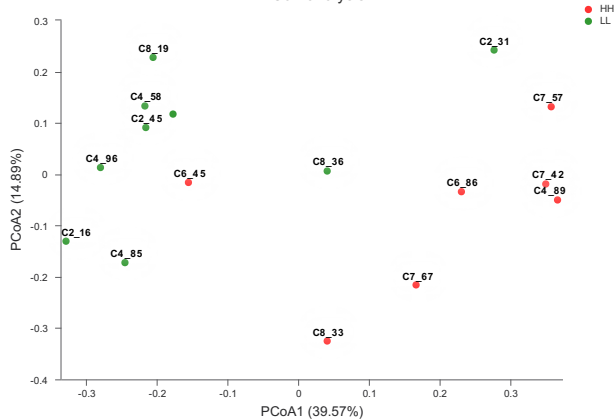

Supplement: Supplementary file 6 — Additional file 5: Figure S2. Microbial compositional profiles of (A) Eukaryota and (B) Viruses of the HH and LL rumen samples visualized using principal-coordinate analysis (PCoA). The first two PCoAs were plotted, and calculated based on the Bray-Curtis dissimilarity matrices at species level. [file 40168_2020_819_MOESM5_ESM.pdf]

A

## Wilcoxon rank-sum test on Phylum level

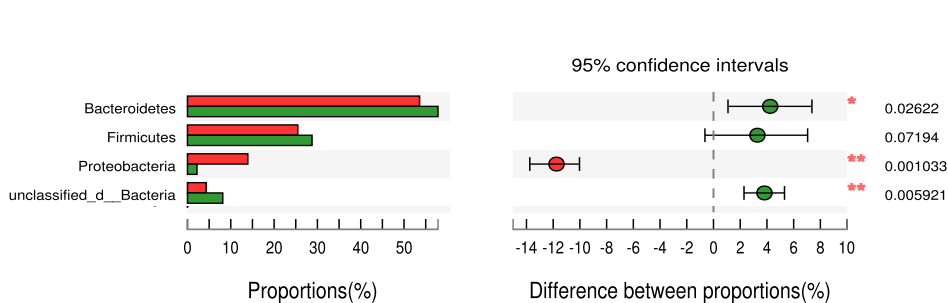

## Wilcoxon rank-sum test on Genus level

B

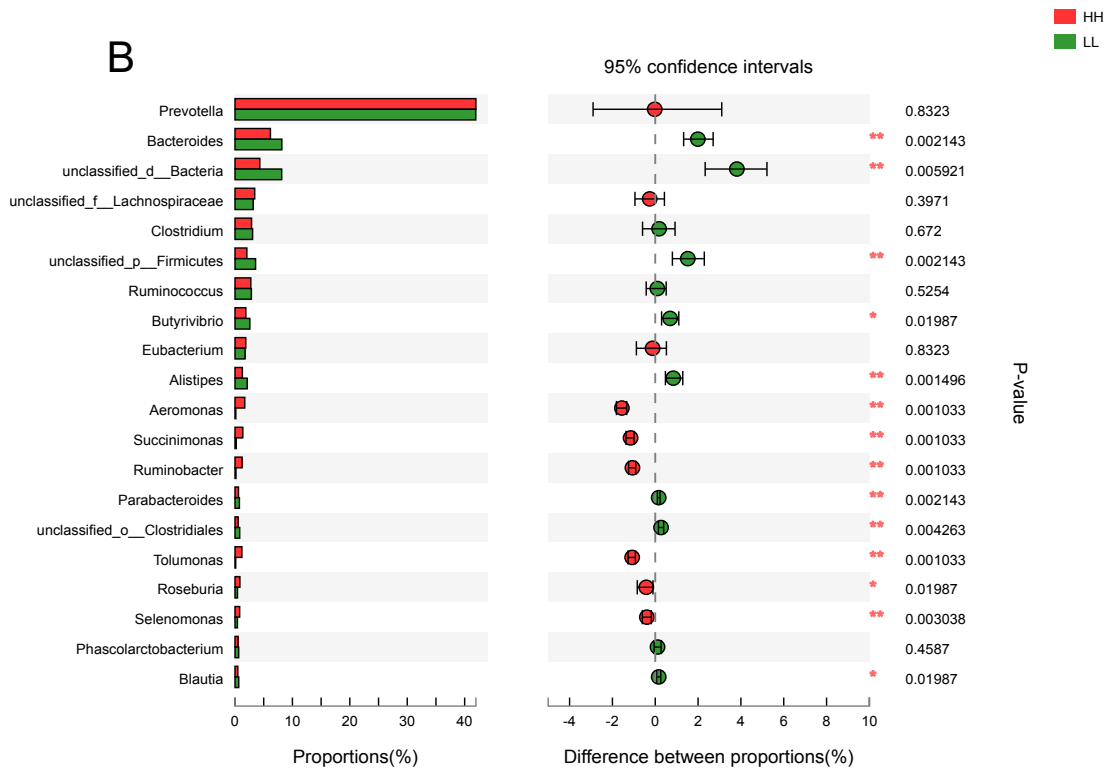

Supplement: Supplementary file 7 — Additional file 6: Figure S3. Comparison of bacterial phyla and genera. Bacterial phyla (A) and genera (B) were tested by Wilcoxon rank-sum test, *P<0.05, **P<0.01. [file 40168_2020_819_MOESM6_ESM.pdf]

A

## Wilcoxon rank-sum test on Phylum level

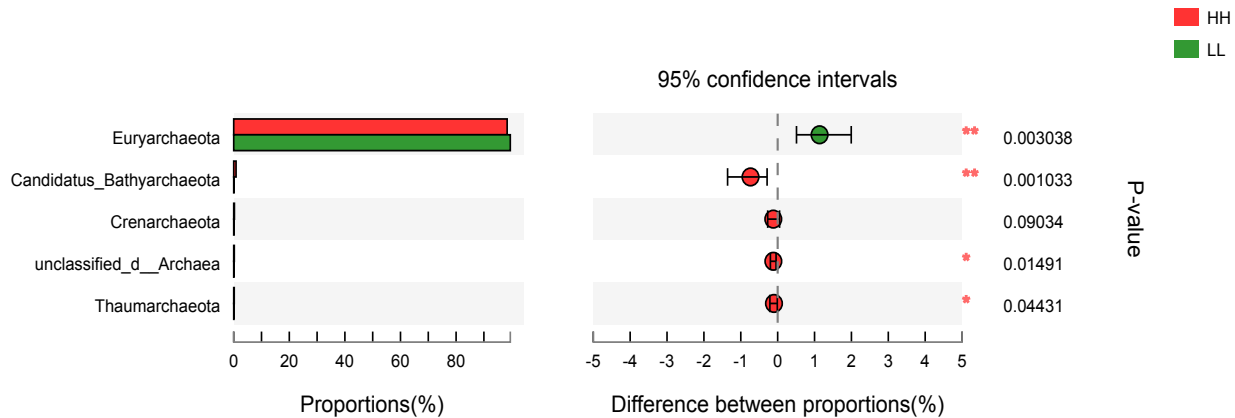

B

## Wilcoxon rank-sum test on Genus level

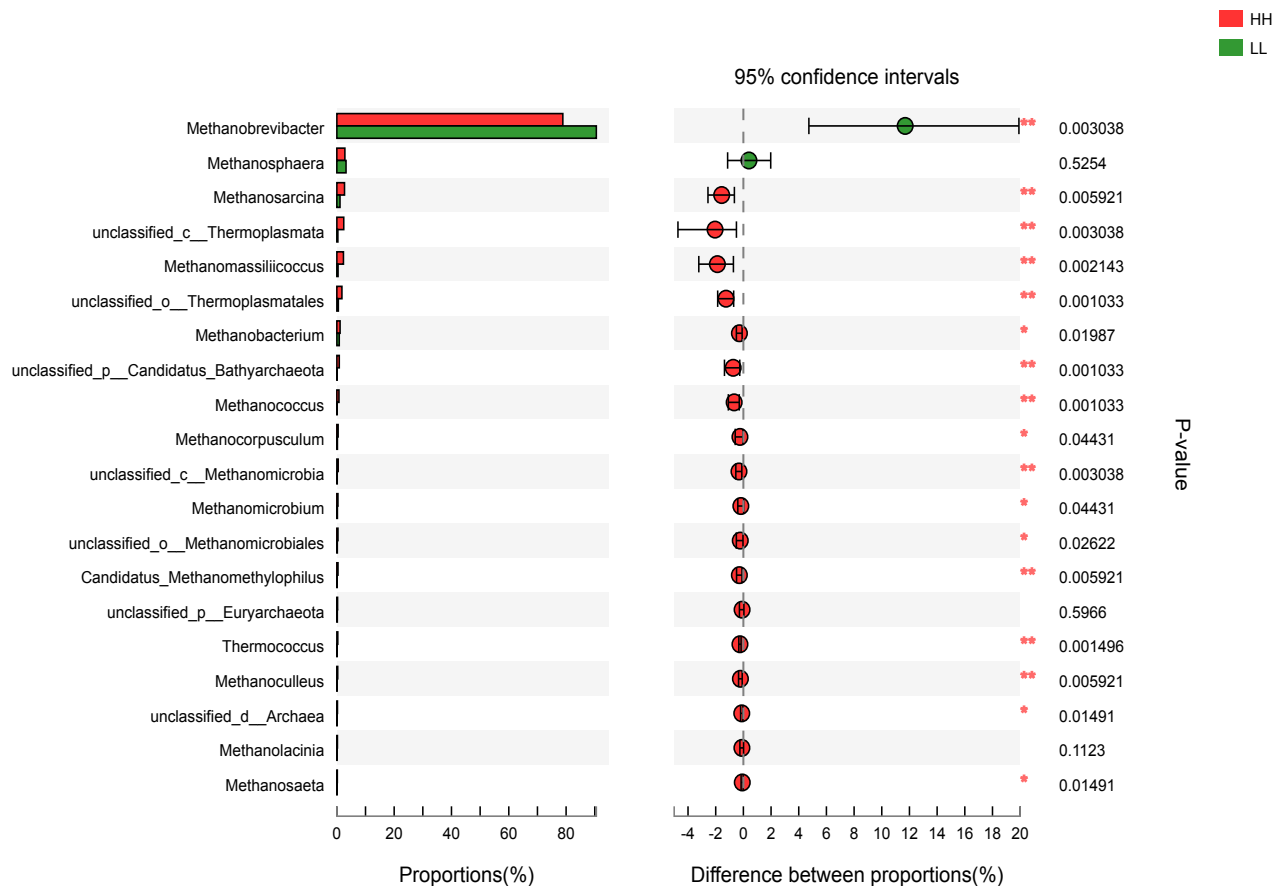

Supplement: Supplementary file 8 — Additional file 7: Figure S4. Comparison of archaeal phyla and genera. Archaeal phyla (A) and genera (B) were tested by Wilcoxon rank-sum test, *P<0.05, **P<0.01. [file 40168_2020_819_MOESM7_ESM.pdf]

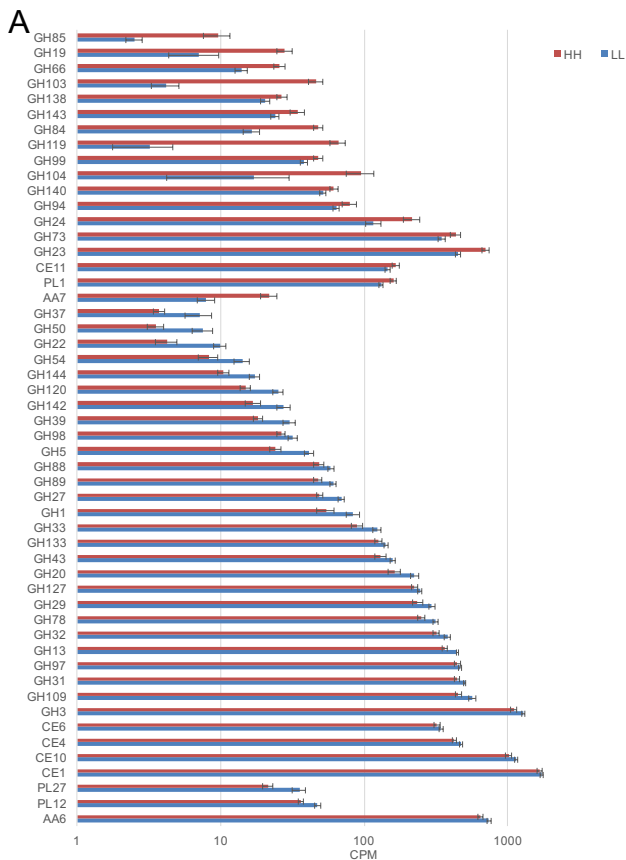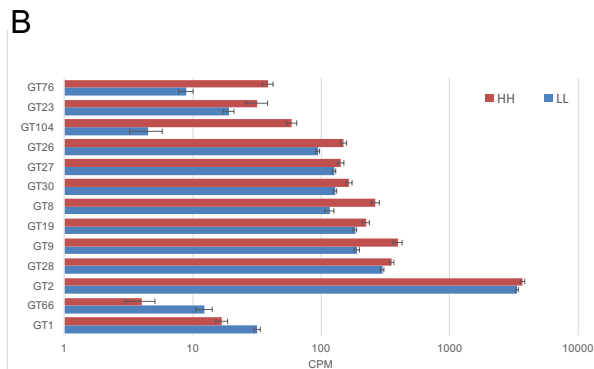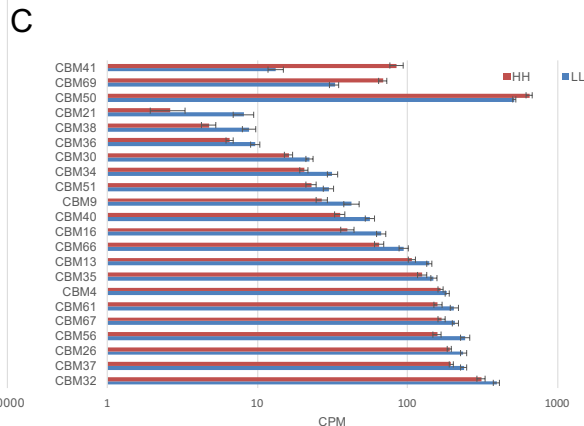

Supplement: Supplementary file 11 — Additional file 10: Figure S5. Differential CAZyme functions between HH and LL cows. (A) Significantly different Glycoside Hydrolases (GHs), Carbohydrate Esterases (CEs), Polysaccharide Lyases (PLs), and Auxiliary Activities (AAs) between the rumen of HH and LL cows. (B) Significantly different GlycosylTransferases (GTs) between the rumen of HH and LL cows. (C) Significantly different Carbohydrate-Binding Modules (CBMs) between the rumen of HH and LL cows. Significantly different CAZymes were tested by Linear discriminant analysis effect size (LEfSe) analysis with linear discriminant analysis (LDA) score of > 2 and P value of < 0.05. [file 40168_2020_819_MOESM10_ESM.pdf]

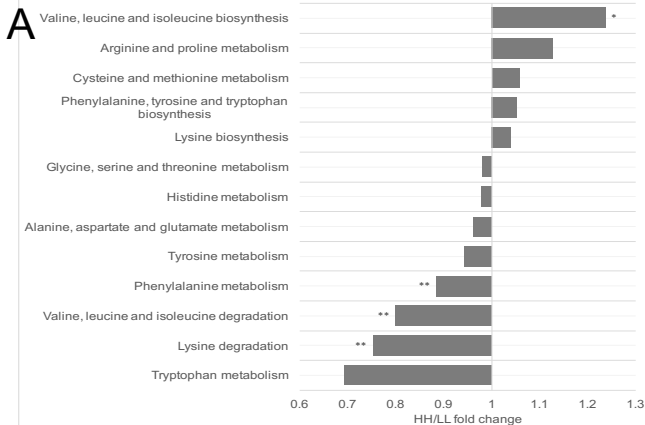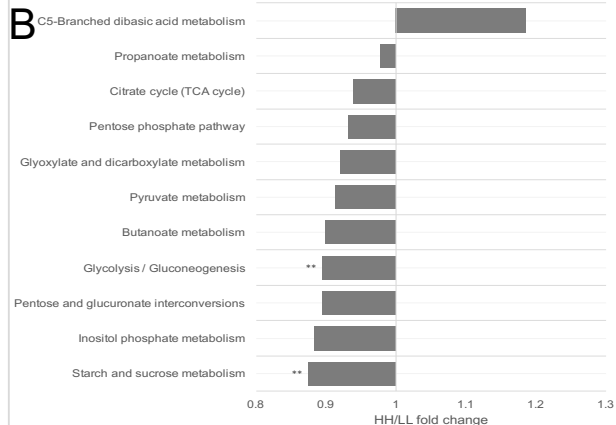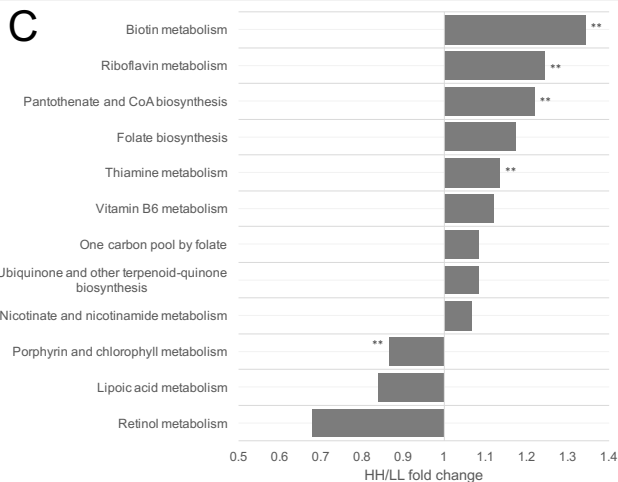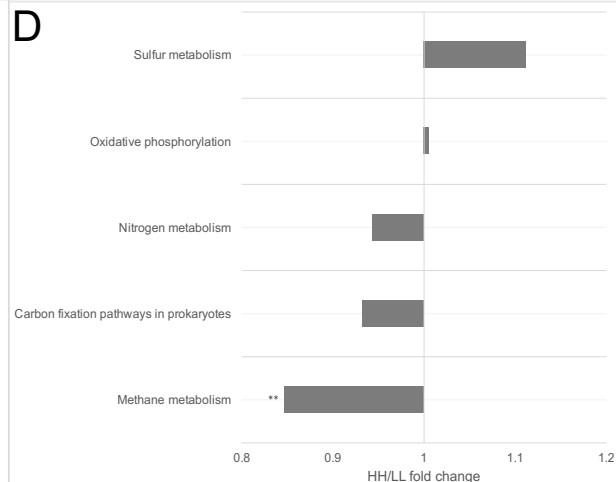

Supplement: Supplementary file 12 — Additional file 11: Figure S6. HH/LL fold change shows differences in level-3 microbial pathways between HH and LL cows. (A) Amino acid metabolism. (B) Carbohydrate metabolism. (C) Metabolism of cofactors and vitamins. (D) Energy metabolism. Significant different pathways were tested by Linear discriminant analysis effect size (LEfSe) analysis with linear discriminant analysis (LDA) score of > 2 and P value of < 0.05. [file 40168_2020_819_MOESM11_ESM.pdf]

A

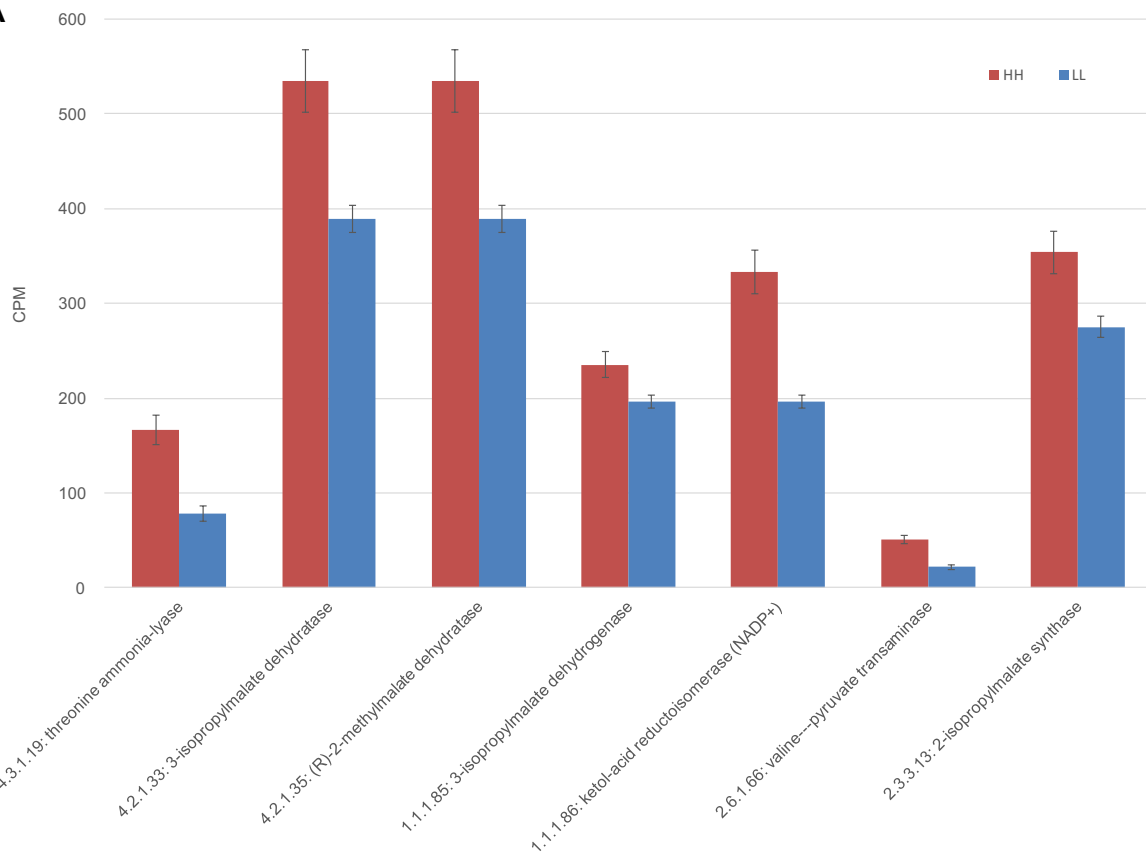

B

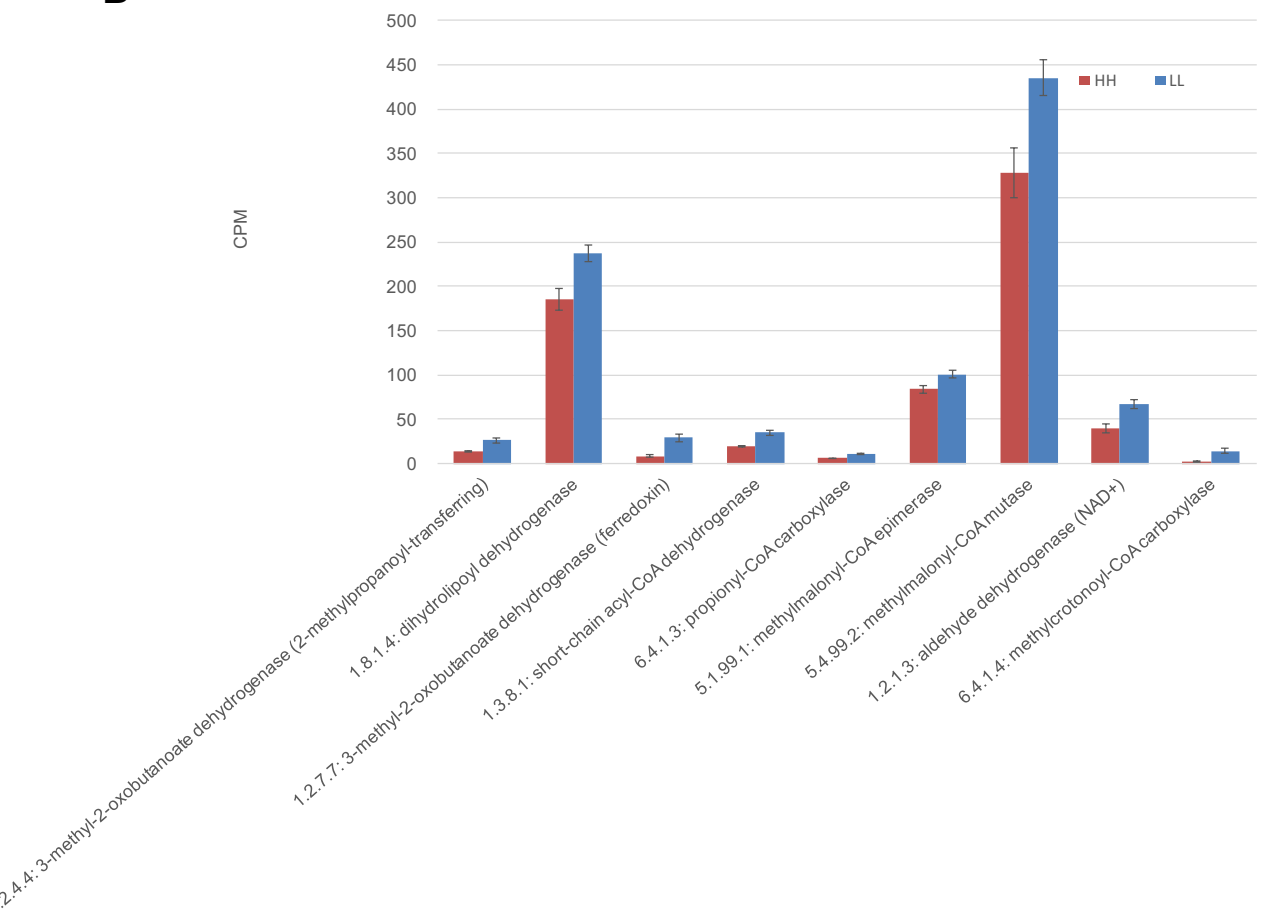

Supplement: Supplementary file 13 — Additional file 12: Figure S7. Comparison of significantly enriched ECs involved in branched chain amino acid biosynthesis (A) and degradation (B). Significantly different pathways were tested by Wilcoxon rank-sum test with adjusted P value of < 0.05. [file 40168_2020_819_MOESM12_ESM.pdf]

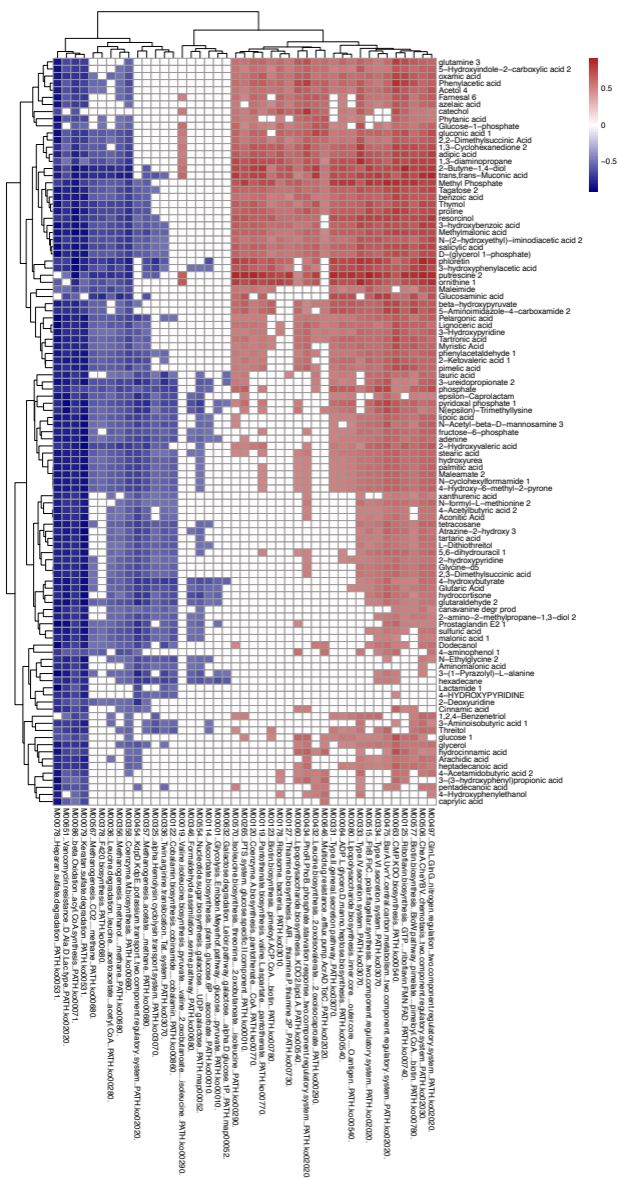

Supplement: Supplementary file 15 — Additional file 14: Figure S8. Association heat map between MPY-positive associated metabotypes and microbiome functional modules. [file 40168_2020_819_MOESM14_ESM.pdf]

A

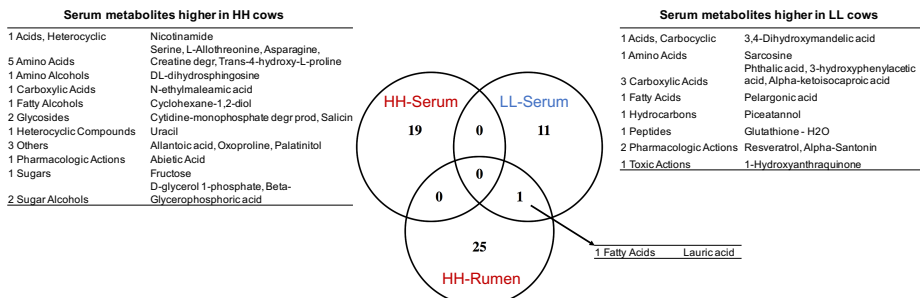

B

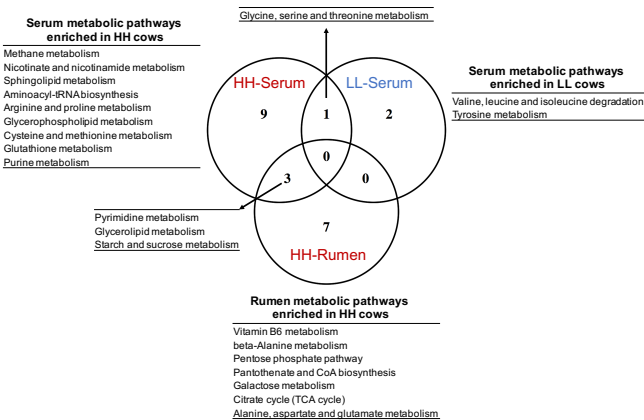

C

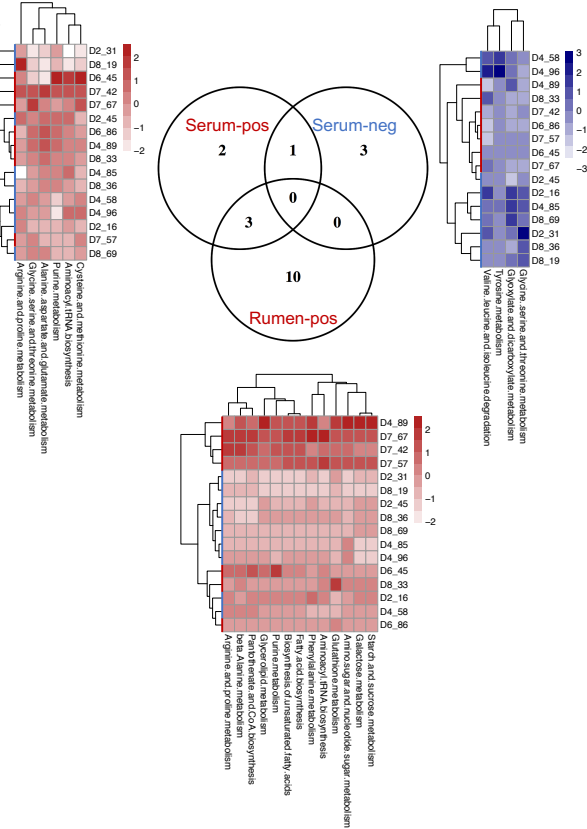

Supplement: Supplementary file 17 — Additional file 16: Figure S9. Comparison of MPY-associated metabolites, metabolites-enriched pathways, and metabolites sets between rumen and serum. (A) The Venn diagram shows significantly different metabolites in rumen and serum between different MPY groups. (B) The Venn diagram shows key pathways (enriched based on the significantly different metabolites). (C) Venn diagram shows MPY-positive and MPY-negative metabolites sets in rumen and serum. Heat maps display the Z score-transformed abundance of each metabolites sets in each sample. [file 40168_2020_819_MOESM16_ESM.pdf]

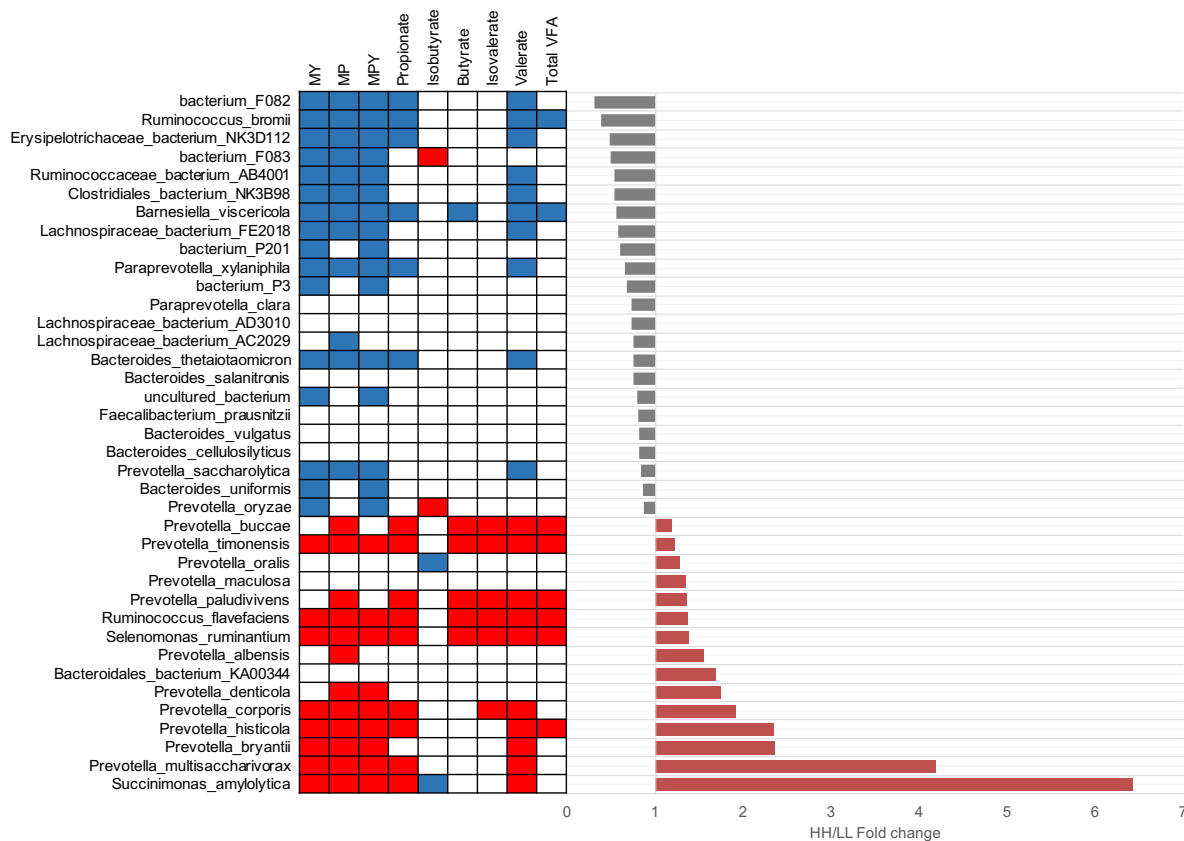

Supplement: Supplementary file 18 — Additional file 17: Figure S10. Associations between significantly enriched bacterial species and metabolites, metabolic pathways, and phenotypes. Only significant correlations (P < 0.05) were presented in the correlation heat map. Red: significantly positive correlations, blue: significantly negative correlations, white: no significant correlation. [file 40168_2020_819_MOESM17_ESM.pdf]
