# Supplementary material for: Multi-omics reveals that the rumen microbiome and its metabolome together with the host metabolome contribute to individualized dairy cow performance
Source: Microbiome. 2020 May 12;8:64. doi: 10.1186/s40168-020-00819-8 (PMC7218573; doi:10.1186/s40168-020-00819-8)
Supplement: Supplementary file 4 — Additional file 3: Figure S1. Profiles of rumen microbial composition of dairy cows. (A) Rumen microbial composition based on the domain-level taxonomy. (B) Bacterial composition based on the phylum-, family-, and genus-level taxonomy. (C) Archaeal composition based on the phylum-, family-, and genus-level taxonomy. (D) Eukaryotic composition based on the kingdom- and phylum-level taxonomy. [file 40168_2020_819_MOESM3_ESM.pdf]

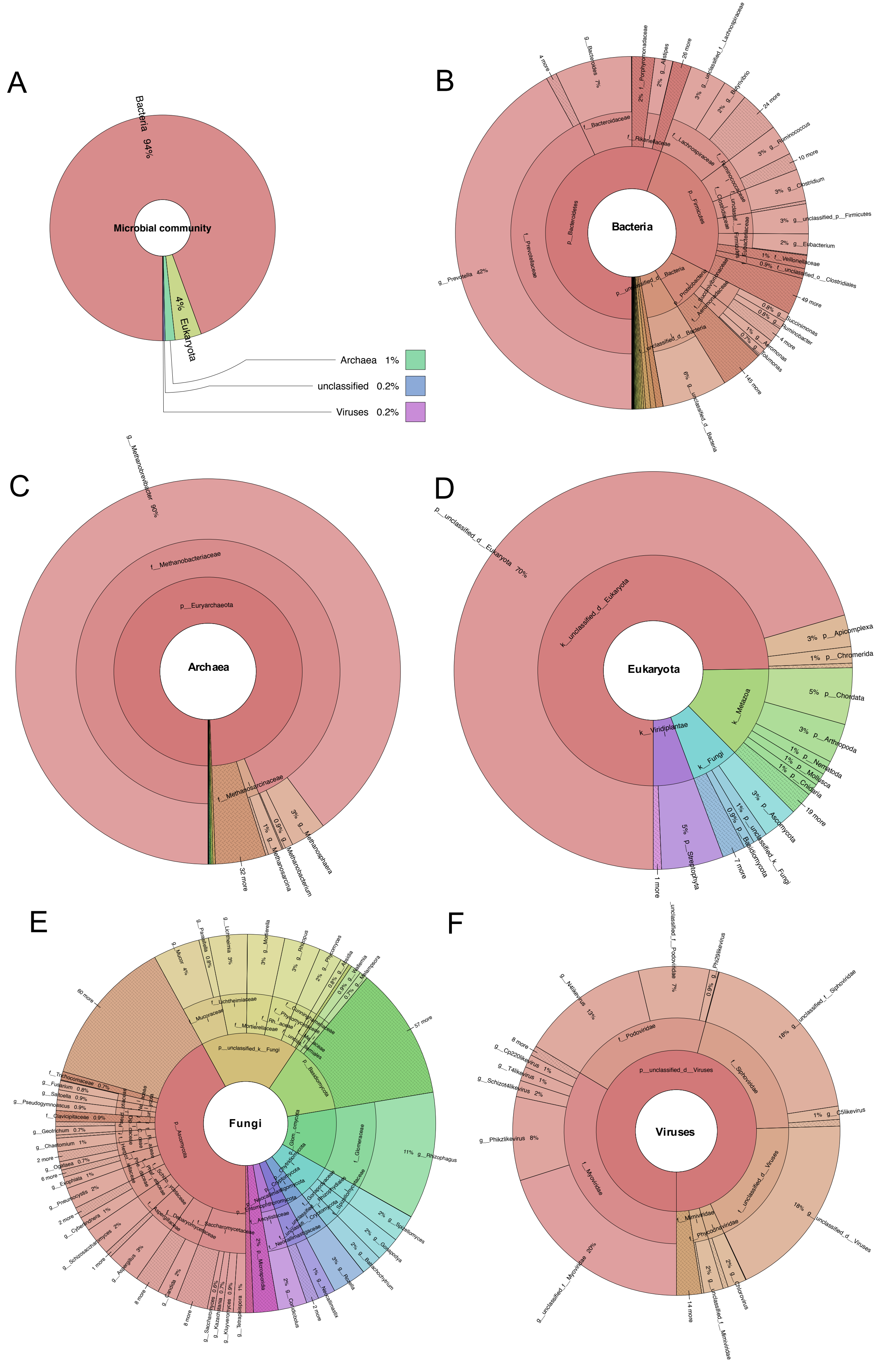
A
Microbial community
Bacteria 94%
4% Eukaryota
Archaea 1%
unclassified 0.2%
Viruses 0.2%
B
Bacteria
p__Bacteroidetes
f__Prevotellaceae
g__Prevotella 42%
f__Bacteroidaceae
g__Bacteroides 7%
4 more
2% f__Porphyromonadaceae
2% g__Alistipes
f__Rikenellaceae
26 more
3% g__unclassified_f__Lachnospiraceae
2% g__Butyrivibrio
24 more
3% g__Ruminococcus
10 more
3% g__Clostridium
3% g__unclassified_p__Firmicutes
2% g__Eubacterium
1% f__Veillonellaceae
0.9% f__unclassified_o__Clostridiales
49 more
0.8% g__Succinimonas
0.8% g__Ruminobacter
4 more
1% g__Aeromonas
0.7% g__Tolumonas
145 more
6% g__unclassified_d__Bacteria
p__Firmicutes
f__Lachnospiraceae
f__Ruminococcaceae
f__Clostridiaceae
f__Eubacteriaceae
p__Proteobacteria
f__Succinivibrionaceae
f__Aeromonadaceae
p__unclassified_d__Bacteria
f__unclassified_d__Bacteria
C
Archaea
p__Euryarchaeota
f__Methanobacteriaceae
g__Methanobrevibacter 90%
f__Methanosarcinaceae
3% g__Methanosphaera
0.9% g__Methanobacterium
1% g__Methanosarcina
32 more
D
Eukaryota
k__unclassified_d__Eukaryota
p__unclassified_d__Eukaryota 70%
3% p__Apicomplexa
1% p__Chromerida
k__Metazoa
5% p__Chordata
3% p__Arthropoda
1% p__Nematoda
1% p__Mollusca
1% p__Cnidaria
19 more
k__Fungi
3% p__Ascomycota
1% p__unclassified_k__Fungi
0.9% p__Basidiomycota
7 more
k__Viridiplantae
5% p__Streptophyta
1 more
E
Fungi
p__Ascomycota
p__unclassified_k__Fungi
p__Basidiomycota
p__Glomeromycota
f__Glomeraceae
11% g__Rhizophagus
57 more
60 more
g__Mucor 4%
g__Parasitella 0.8%
g__Lichtheimia 3%
3% g__Mortierella
3% g__Rhizopus
2% g__Phycomyces
0.8% g__Absidia
0.9% g__Waltemia
0.7% g__Melampsora
f__Mucoraceae
f__Lichtheimiaceae
f__Mortierellaceae
f__Cunninghamellaceae
f__Phycomycetaceae
f__Trichocomaceae 0.7%
g__Fusarium 0.8%
g__Saitoella 0.9%
g__Pseudogymnoascus 0.9%
f__Clavicipitaceae 0.9%
g__Geotrichum 0.7%
g__Chaetomium 1%
2 more
g__Ogataea 0.7%
6 more
g__Exophiala 1%
g__Pneumocystis 2%
2 more
g__Cyberlindnera 1%
g__Schizosaccharomyces 2%
1 more
g__Aspergillus 3%
8 more
g__Candida 2%
8 more
g__Saccharomyces 0.6%
g__Kazachstania 0.7%
g__Kluyveromyces 0.9%
g__Tetrapisispora 1%
f__Aspergillaceae
f__Debaryomycetaceae
f__Saccharomycetaceae
p__Microsporidia
p__Neocallimastigomycota
p__Entomophthoromycota
p__Chytridiomycota
p__Cryptomycota
f__Ancylistaceae
f__Neocallimastigaceae
f__Gonapodyaceae
f__Spizellomycetaceae
2% g__Conidiobolus
2 more
1% g__Neocallimastix
3% g__Rozella
2% g__Batrachochytrium
2% g__Gonapodya
2% g__Spizellomyces
F
Viruses
p__unclassified_d__Viruses
f__Podoviridae
f__Siphoviridae
f__Myoviridae
f__unclassified_d__Viruses
f__Mimiviridae
f__Phycodnaviridae
g__N4likevirus 13%
g__unclassified_f__Podoviridae 7%
0.9% g__Phi29likevirus
18% g__unclassified_f__Siphoviridae
1% g__C5likevirus
8 more
g__Cp220likevirus 1%
g__T4likevirus 1%
g__Schizot4likevirus 2%
g__Phikzlikevirus 8%
18% g__unclassified_d__Viruses
g__unclassified_f__Myoviridae 20%
14 more
2% g__unclassified_f__Mimiviridae
2% g__Chlorovirus
